# The role of ion migration, octahedral tilt, and the A-site cation on the instability of Cs_1-x_FA_x_PbI_3_

**DOI:** 10.1038/s41467-023-44235-6

**Published:** 2023-12-22

**Authors:** Weilun Li, Mengmeng Hao, Ardeshir Baktash, Lianzhou Wang, Joanne Etheridge

**Affiliations:** 1 School of Physics and Astronomy, Monash University, Clayton, VIC 3800 Australia; 2 Australian Institute for Bioengineering and Nanotechnology, The University of Queensland, St Lucia, QLD 4072 Australia; 3 School of Chemical Engineering, The University of Queensland, St Lucia, QLD 4072 Australia; 4 Monash Centre for Electron Microscopy, Monash University, Clayton, VIC 3800 Australia; 5 Department of Materials Science and Engineering, Monash University, Clayton, VIC 3800 Australia

**Keywords:** Solar cells, Quantum dots

## Abstract

Organic-inorganic hybrid perovskites are promising materials for the next generation photovoltaics and optoelectronics; however, their practical application has been hindered by poor structural stability mainly caused by ion migration and external stimuli. Understanding the mechanism(s) of ion migration and structure decomposition is thus critical. Here we observe the sequence of structural changes at the atomic level that precede structural decomposition in the technologically important Cs_1-x_FA_x_PbI_3_ using ultralow dose transmission electron microscopy. We find that these changes differ, depending upon the A-site composition. Initially, there is a random loss of FA^+^, complemented by the loss of I^-^. The remaining FA^+^ and I^-^ ions then migrate, unit cell by unit cell, into an ordered and more stable phase with a √2 x √2 superstructure. Further ion loss is accompanied by A-site dependent octahedral tilt modes and associated tetragonal phases with different stabilities. These observations of the loss of FA^+^/I^-^ ion pairs, ion migration, octahedral tilt modes, and the role of the A-cation, provide insights into the atomic-scale structural mechanisms that drive and block ion loss and ion migration, opening pathways to inhibit ion loss, migration and improve structural stability.

## Abstract (web summary)

Applications of perovskite solar cells have been hindered by ion migration and structural instability. Here, the authors use ultra-low dose transmission electron microscopy to reveal the process of ion migration and structural change at the atomic level in Cs_1-x_FA_x_PbI_3_.

## Introduction

Organic–inorganic hybrid perovskites (OIHPs) are promising materials for applications, including photovoltaics (PVs)^1–4^, light-emitting diodes (LEDs)^5,6^ and lasers^7,8^. They have a common ABX_3_ structure (where A = organic cations, such as methylammonium (MA^+^) and formamidinium (FA^+^) or inorganic Cs^+^; B = Ge^2+^, Sn^2+^, Pb^2+^; and X = halogen). This flexible structure with A-site cations surrounded by the corner-sharing [BX_6_]^4−^ octahedral framework is thought to underpin the excellent power conversion efficiencies^9^, long carrier diffusion lengths^10^ and tuneable band gap^11^ of OIHPs. However, several critical limitations on performance remain, including ion migration^12^, current–voltage hysteresis^13,14^ and material degradation in air and light^15,16^. Substantial efforts have been devoted to understanding correlations between the microstructure and physical properties of OIHPs, thus finding strategies to improve their performance. However, an in-depth understanding of their structure and defect structure at the atomic level is still lacking. In particular, there are outstanding questions regarding how and where vacancies are formed and their role in ion migration, hysteresis, structural change, and ultimately decomposition.

Transmission electron microscopy (TEM) is a powerful tool for revealing the local atomic structure of materials, however, the pristine structure of OIHPs can be readily modified by the electron beam^17^. This presents challenges but also opportunities. Although extremely challenging, TEM data can be obtained from pristine structures if careful protocols for TEM specimen preparation, transfer and examination are applied, including ensuring the total electron dose lies well below measured damage thresholds. Under these controlled circumstances, there is an opportunity. While the physical mechanism of damage with light and electrons may be different, electron-induced damage can act as a proxy to provide insights into possible pathways for structural degradation. With careful control of additional electron dose above damage thresholds, sequences of structural change can be observed, revealing possible mechanisms for vacancy formation, ion migration, phase transitions and ultimately structural decomposition.

This approach has been applied variously to the pure ABX_3_ systems, MAPbI_3_, MAPbBr_3_ and FAPbI_3_, either in the form of bulk thin-film or quantum dots or nanocrystals. The structural response in these systems has been shown to be the same, irrespective of whether they are in quantum dot or bulk form^18–20^. In all cases, it has been observed that the final decomposition product is BX_2_ (e.g., PbI_2_ or PbBr_2_)^18–24^ or Pb^21,25,26^. However, the initial perovskite ABX_3_ structures do not collapse directly into BX_2_ but form an intermediate phase^18–20,23–25^.

In the case of FAPbI_3_, an intermediate phase was observed recently using moderately low dose atomic resolution scanning transmission electron microscopy (STEM) (dose ~200 e/Å^2^/s)^20^. This revealed a √2 × √2 ordered structure of FA^+^ vacancies (V^−^_FA_) in the decomposition from cubic FAPbI_3_ into PbI_2_. This specific superstructure stabilized by ordered A-site vacancies has been proposed to explain the unusual regenerative properties of hybrid perovskite solar cells when degraded MAPbI_3_ solar cells are post-treated with gaseous MAI^27^.

In the case of MAPbX_3,_ intermediate phases were first detected from the appearance of additional (sometimes called forbidden) reflections in selected area diffraction (SAD) patterns (~1–2 e/Å^2^/s)^18,23,24,28^. Different structural models were proposed for these intermediate phases, such as ordered halogen vacancies^18,24,28^ and octahedral tilt or rotation^23^, however, these models cannot be distinguished easily from SAD alone. Recently, further information was obtained for the specific case of MAPbI_3_ using low-dose high-resolution TEM (HR-TEM) combined with a direct-detection electron counting camera (DDEC) which observed an intermediate phase of MA_0.5_PbI_3_ similar to that observed in FAPbI_3_^19^.

Despite these important observations, the mechanisms underpinning structural instability and ion migration remain unclear, including the mechanism by which ordered A-site vacancies are formed and whether there is any associated ordering of I^−^ vacancies (V^+^_I_) and/or octahedral tilting. Furthermore, we need to understand what role, if any, the A-cation might play, as A-site engineering may provide an avenue for improving device stability^29^. We investigate these questions in the present paper for the technologically important mixed-cation perovskite Cs_1−*x*_FA_*x*_PbI_3_. Such mixed-cation perovskites (A_1−*x*_A’_*x*_BX_3_) have attracted great interest in photovoltaic applications due to their relatively good stability and charge transport properties^30–32^. The crystal structure of bulk and quantum dot Cs_1−*x*_FA_*x*_PbI_3_ is the same, so either is suitable for this study^33–36^. We choose to examine quantum dots because they align consistently along a major zone axis, facilitating minimization of electron dose. We examine a high-quality synthesis that previously achieved a certified record power conversion efficiency of 16.6% at a composition of Cs_0.5_FA_0.5_PbI_3_^37^. We also note that quantum dots have their own exciting applications in photo-active devices^37,38^.

We first examine the pristine structures for pure FAPbI_3_ and Cs_0.5_FA_0.5_PbI_3_, and then the subsequent structural evolution at the atomic level, through ion-vacancy formation and ion migration, using ultra-low-dose HR-TEM with DDEC and low-dose annular dark-field STEM (STEM-ADF).

## Results

### Intermediate phase 1: A-cation and halogen vacancies (V^−^_A_ and V^+^_I_) and ordering

**Fig. 1:**
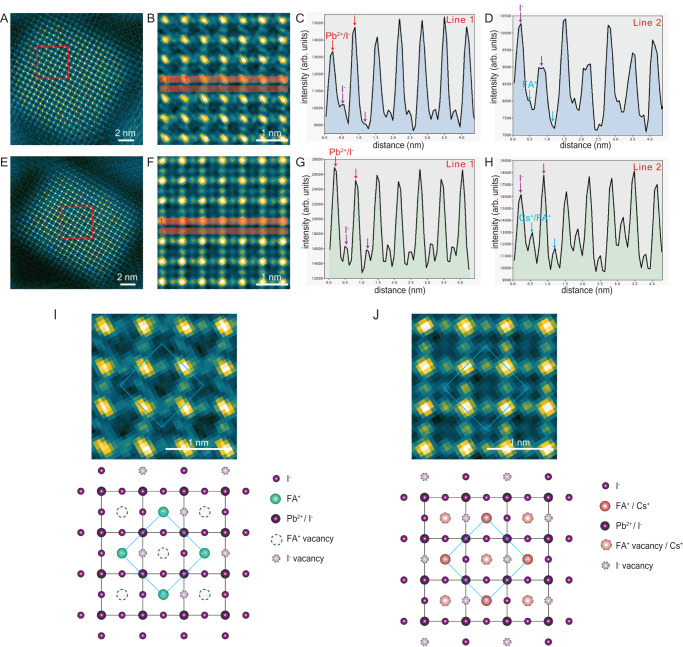
A-site and I^−^ vacancy ordering in FAPbI_3_ and Cs_0.5_FA_0.5_PbI_3_- Low dose STEM-ADF images in the 〈001〉zone axis. **A**–**D** FAPbI_3_. **E**–**H** Cs_0.5_FA_0.5_PbI_3_. **A**, **E** Lower-magnification images. Total dose is 44 e/Å^2^. **B**, **F** Enlarged images of regions marked in (**A**, **E**). **C**, **G** Intensity line profiles integrated over Pb^2+^/I^−^ and I^−^ columns as marked in region 1 in (**B**, **F**). **D**, **H** Intensity line profiles integrated over I^−^ and FA^+^ (or Cs^+^/FA^+^, in the case of Cs_0_^.^_5_FA_0.5_PbI_3_) columns as marked in region 2 in (**B**, **F**). Arrows highlight atomic columns: red—Pb^2+^/I^-^; purple—I^−^; blue—FA^+^ (or Cs^+/^FA^+^). **I**, **J** schematic diagrams of vacancy ordering in FAPbI_3_ and Cs_0.5_FA_0.5_PbI_3_. Blue diamonds indicate the ordered remaining A-site cations. Low-dose STEM-ADF images are filtered by a combined Bragg filter and Butterworth filter to enhance image contrast. All structures and orderings present in the filtered images were also observed to exist in the raw images (details and raw images in Supplementary Note 4). Source data are provided as a Source Data file.

To identify unambiguously any atomic-level compositional variations, we first use low-dose STEM-ADF because the image intensity is directly related to the number and species of atoms in the atomic column (Fig. 1). The total electron dose was carefully minimized and measured by a direct electron detector to be 44 e/Å^2^ per image (Supplementary note 1). This dose was the lowest we could use while still obtaining interpretable STEM-ADF images with atomic-level information (that is, sufficient signal-to-noise ratio (SNR)). However, we note that while this dose is several orders of magnitude lower than conventional STEM-ADF images, it still represents a significant dose for this class of materials, and we anticipate some electron-beam damage.

We consider first FAPbI_3_, Fig. 1A–D. The pristine structure has previously been determined to be cubic (space group: ${pm}\bar{3}m$) using synchrotron x-ray diffraction (XRD)^37,39^. In the 〈001〉zone axis atomic-resolution STEM-ADF image, the highest intensity maxima correspond to atomic columns comprising Pb^2+^/I^−^ with small local maxima in-between corresponding to I^−^ columns (see intensity line scan along 〈100〉, Fig. 1C); the middle-intensity maxima correspond to I^−^ columns and the lowest intensity maxima correspond to FA^+^ columns due to less scattering to high angles from the organic molecule (Fig. 1B). In this image, it is evident that the image intensity at the FA^+^ column positions alternates (high–low–high–low), suggesting a doubling of the original cubic unit cell. This is confirmed by the intensity line profile across the FA^+^ column positions (Fig. 1D). Moreover, we also observe similar ordering of the intensity at the Pb^2+^/I^−^ columns and I^-^ columns (Fig. 1C, D). These lower-intensity FA^+^ columns and I^−^ columns suggest the presence of vacancies. Moreover, an ordered pattern of both FA^+^ and I^−^ vacancies is evident, making a coordinated √2 × √2 superstructure of V^-^_FA_ and V^+^_I_ (Fig. 1I). We have confirmed this image interpretation using STEM-ADF image simulations. In particular, simulations show that the coordinated intensity modulations at the FA^+^ and Pb^2+^/I^−^ and I^−^ sites are not due to the effects of dynamical electron scattering. (supplementary figure 1).

We note in passing that despite the evident superstructure in the STEM images, the image SNR is too low to generate any detectable superlattice reflections in the corresponding Fourier transform (FT). Hence, we do not use FTs as a method to identify the minimum dose at which damage occurs (Supplementary Note 3). We also confirm that these vacancies and ordering are evident in the lowest dose raw data images and are not introduced by the post-filtering process (Supplementary Note 4).

Let us now consider the Cs_0.5_FA_0.5_PbI_3_ (Fig. 1E–H). In this case, the A-site contains a mix of FA^+^ and Cs^+^ with the pristine structure previously being determined to be cubic (space group: ${pm}\bar{3}m$)^37^. As with FAPbI_3_, we observe an alternating modulation of image intensity at all three atomic column sites (Pb^2+^/I^−^, I^−^ and at the A-site, in this case, FA^+^/Cs^+^ (Fig. 1E, F). Interestingly, the intensity line profiles indicate a smaller difference between high-intensity A-site columns and low-intensity A-site columns than for FAPbI_3_, Fig. 1G, H. This suggests fewer FA^+^/Cs^+^ and I^−^ vacancies have occurred in the mixed cation Cs_0.5_FA_0.5_PbI_3_, compared with FAPbI_3_ for the same electron dose. For Cs_0.5_FA_0.5_PbI_3_, the A-site columns are nominally half occupied by FA^+^ and half by Cs^+^. We hypothesize that FA^+^ vacancies occur more readily than Cs^+^ vacancies (because FA^+^, is known to readily break down into smaller molecules (such as NH_3_ and CH_2_N)^19,40^), so while FA^+^ cations may be lost, Cs^+^ cations remain on the A-site in sufficient numbers to generate intensity peaks in the ADF image. Hence, lower intensity peaks are still visible in the image at the vacancy-containing FA^+^/Cs^+^ columns, due to the remaining Cs^+^ cations (Fig. 1J and Supplementary Note 5). Whereas for FAPbI_3_, the lower intensity ‘peaks’ at the vacancy-containing A-site columns are barely visible or invisible.

To summarize the observations thus far, a low electron dose of 44 e/Å^2^ applied with a scanned focussed electron probe, is sufficient to induce an ordered and coordinated √2 × √2 superstructure of A-site and I^−^ vacancies in both FAPbI_3_ and Cs_0.5_FA_0.5_PbI_3._ Furthermore, image contrast is consistent with the A-site vacancies in Cs_0.5_FA_0.5_PbI_3_ being predominantly FA^+^ cations, rather than Cs^+^.

### Formation mechanism—intermediate phase 1−FA^+^/I^−^ ion migration and ordering in FAPbI_3_

A key question arises, namely, how do these ordered A-site and I^−^ vacancy superstructures form from the initial undamaged cubic phase. We address this question by examining FAPbI_3_ using an even lower dose imaging method, namely phase contrast HR-TEM combined with a DDEC. This technique can be performed with an order of magnitude lower dose than that of STEM-ADF and offers much better temporal resolution. However, the image contrast mechanism is different from STEM-ADF and the relationship between atomic column composition and image contrast is less direct. For this reason, we only consider images of FAPbI_3_ where the A-site only comprises FA^+^, so intensity variations at the A-site can be exclusively related to the occupancy of FA^+^. With the DDEC, we obtained successive images, each with a dose of 1.5 e/Å^2^ and then we summed sequences of these to provide images corresponding to a dose of our choice. This allows an identification of the dose at which structural changes begin and, critically, allows us to observe the subsequent structural changes, step-by-step, at the atomic level. We note that due to the different illumination conditions in STEM-ADF and in HR-TEM and the different way of estimating electron dose, the absolute dose may not be directly comparable and the critical dose for inducing structural changes may be different across the two techniques.

In the first instance, we sum six successive HR-TEM image frames, each acquired with a dose of ~1.5 e/Å^2^, to form an image where the atomic structure can just be resolved with sufficient signal-to-noise ratio to permit quantitative measurements (Supplementary Notes 6 and 7). In the raw image, the FA^+^ columns appear to have uniform image intensity and the corresponding FT is consistent with the pristine cubic perovskite structure, with no additional reflections. However, in the integrated column intensity map, some intensity variations are revealed (even though the precision of the column intensity analysis is affected by shot noise at such a low dose (7.8 e/Å^2^)). These variations are consistent with the presence of random V^−^_FA_ and V^+^_I_. Occasionally, there are even ordered vacancies in some local regions. Given the low dose, it could be that these vacancies are intrinsic to the pristine FAPbI_3_, particularly if prepared with insufficient surface ligand (oleic acid). Or it could be that, even at this low dose, there are sufficient electrons to generate a few vacancies. Both explanations may apply. However, the number of vacancies that were observed at this stage (HR-TEM at 7.8 e/Å^2^) is much less than in the first acquired STEM-ADF images taken with 44 e/Å^2^ (Fig. 1). This suggests at least some of the vacancies observed in the first STEM-ADF images (Fig. 1) were induced by the electron beam, even though the STEM-ADF image was taken with the lowest achievable dose for STEM-ADF. We cannot know whether the vacancies incurred in the HR-TEM (taken at 7.8 e/Å^2^) are intrinsic to the specimen or induced by the electron beam. We hypothesize that it is likely both are true.

**Fig. 2:**
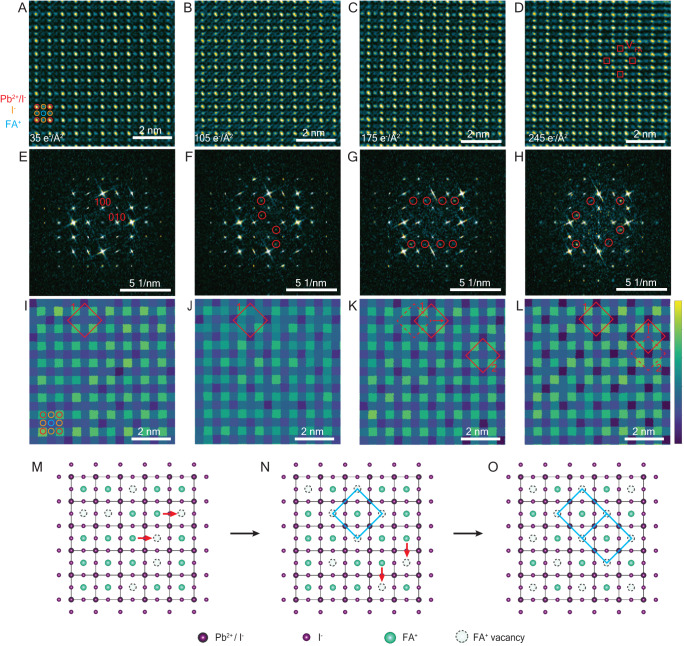
Atomic-scale HR-TEM images of FAPbI_3_ show the initial random FA^+^ vacancies and subsequent ordering via FA^+^ ion migration. **A**–**D** HR-TEM images of FAPbI_3_ with increasing electron beam exposure. Red circles correspond to the highest intensity Pb^2+^/I^−^ columns; orange circles correspond to I^−^ columns and blue circles correspond to the FA^+^ column. Red squares in (**D**) highlight ordered V^−^_FA_. **E**–**H** FTs of images (**A**–**D**). Forbidden $1/2,1/2,0$ and $1/2,3/2,0$ reflections are marked by red circles. **I**–**L** Integrated column intensity maps based on HR-TEM images in (**A**–**D**). Colour bar represents integrated intensity from 5000 to 10,000 in arb. units. Red solid diamonds highlight ordered V^−^_FA_ pattern in the image and red dashed diamonds highlight ordered V^−^_FA_ pattern in the previous image. **M**–**O** schematic diagrams illustrating the process of loss, migration and ordering of FA^+^ revealed in (**A**–**D**) and (**I**–**L**). Selected regions of ordered V^−^_FA_ are marked by blue diamonds. Red arrows indicate the migration of FA^+^. Black arrows between **M**, **N** and **N**, **O** indicate the loss and migration of ions with time series (or dose).

To improve the SNR, we sum additional frames to generate an image corresponding to a total dose of 35 e/Å^2^. The same observations apply as to the 7.8 e/Å^2^ images but with greater clarity (Fig. 2I). At this stage, vacancies are still largely random and have not been ordered in a √2 × √2 superstructure, confirming there is a random initial loss of FA^+^ and I^−^ pairs. With a further increase in the total dose (105 e/Å^2^), the contrast in the image changes, most evident at FA^+^ column positions (Fig. 2B). In the corresponding Fourier transform, additional $1/2,1/2,0$_c_ and $1/2,3/2,0$_c_ reflections are evident, inconsistent with the initial cubic structure with a space group ${pm}\bar{3}m$ (Fig. 2F). The integrated intensity map (Fig. 2J) shows significant variations in the FA^+^ column intensity and in some regions, these have formed into an ordered pattern (e.g., region 1). With further continuous beam exposure (to 175 e/Å^2^ and then 245 e/Å^2^), V^−^_FA_ and its ordering can be observed directly in the HR-TEM images (Fig. 2C, D). In the FT, the number of forbidden reflections also increases, and the intensity of forbidden reflections becomes stronger (Fig. 2G, H). Most interestingly, the formation of a fully ordered √2 × √2 pattern of V^−^_FA_ vacancies is clearly visualized in the corresponding column intensity maps (Fig. 2K, L).

These HR-TEM image series reveal a step-by-step process of migration of FA^+^ ions via A-site vacancies to form and an ordered √2 × √2 superstructure. For example, in region 1 from Fig. 2I, J, an ordered square of V^−^_FA_ is formed. This square of vacancies further diffuses by one unit cell (Fig. 2J and K), so the intensity is reversed (i.e., low-intensity FA^+^ vacancy columns become high-intensity FA^+^ occupied columns and vice versa). The same V^−^_FA_ diffusion process is observed in region 2 (Fig. 2K and L), resulting in a fully ordered pattern across the combined region. Our observations here suggest that the initial loss of FA^+^ cations is random, and an ordered pattern is formed by the subsequent migration of FA^+^ ions via V^−^_FA_. This demonstrates the mechanism of loss, migration, and ordering of FA^+^ and is schematically illustrated in Fig. 2M–O.

In parallel with the above analysis of FA^+^ vacancies, we performed a similar analysis of the intensity change of Pb^2+^/I^−^ and I^−^ columns to study the presence of I^−^ vacancies (Supplementary Note 8). Consistent with the observations from ADF-STEM, we find that the I^-^ vacancies are correlated with the FA^+^ vacancies. In addition, and significantly, we observe the same process of migration of iodine ions via V^+^_I_ to form a √2 × √2 V^+^_I_ superstructure. This appears to occur in consort with the FA^+^ vacancies and the formation of the √2 × √2 V^−^_FA_ superstructure.

### Intermediate phases 2: A-site dependent octahedral tilting in FAPbI_3_ and Cs_0.5_FA_0.5_PbI_3_—initial observations

Thus far, we have observed the initial FA^+^/I^−^ vacancies and examined the associated FA^+^/I^−^ ion migration to form an ordered √2 × √2 V^−^_FA_ and V^+^_I_ vacancy superlattice—the intermediate phase 1. We now investigate whether there are any subsequent structural changes with further exposure to the electron beam, before the established final decomposition to PbI_2_. The first phase change, the ordered pattern of vacancies, was evident in ADF-STEM after the first scan at 44 e/Å^2^ (and at lower doses in HR-TEM). We now examine a sequence of subsequent ADF-STEM images of FAPbI_3_ and Cs_0.5_FA_0.5_PbI_3_ taken with increasing electron dose.

**Fig. 3:**
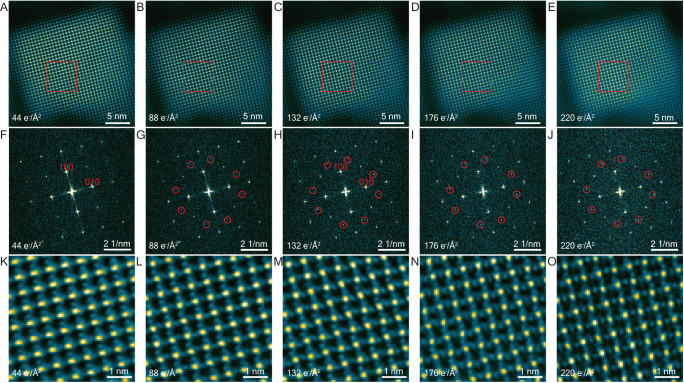
Sequence of STEM-ADF images of FAPbI_3_ shows the structural change of octahedral framework. **A**–**E** STEM-ADF images with increasing total dose. **F**–**J** FTs of STEM-ADF images in (**A**–**E**). Reflections forbidden in the cubic structure are highlighted by red circles. **K**–**O** enlarged images of the regions marked in (**A**–**E**).

In the case of FAPbI_3_ (Fig. 3), the first scan (at ~44 e/Å^2^) exhibits the beginnings of a √2 × √2 V^−^_FA_ and V^+^_I_ vacancy superstructure (just as we found in Fig. 1, confirmed by intensity line profiles in Supplementary Note 9). In the second scan (at ~88 e/Å^2^), very weak additional $1/2,3/2,0$_c_ reflections (and their symmetry-equivalents, highlighted by red circles) appear in the FT (Fig. 3B, at 88 e/Å^2^). After three scans (Fig. 3C, at 132 e/Å^2^), these reflections are much stronger. Moreover, a distortion of the perovskite framework is clearly evident in the zoom-in image (Fig. 3M), consistent with octahedral tilting (see later). We expose for a further two scans and find there are no newly formed reflections nor any additional structural changes (Fig. 3D, E). We will call this, FAPbI_3_—intermediate phase 2. (Note that this is not fully stoichiometric due to the loss of FA^+^/I^−^.)

**Fig. 4:**
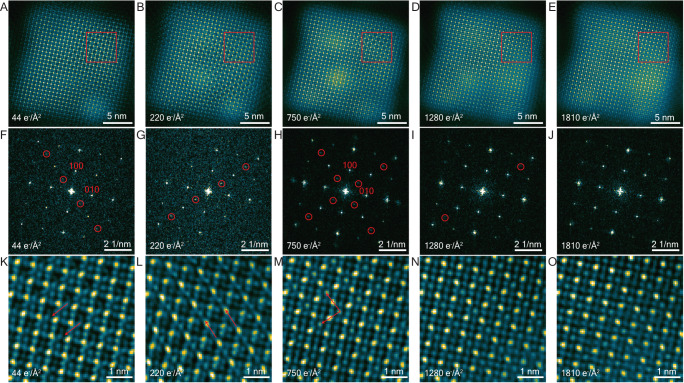
Sequence of STEM-ADF images of Cs_0.5_FA_0.5_PbI_3_ shows a structural change of octahedral framework that is different from FAPbI_3_. **A**–**E** STEM-ADF images with increasing total dose. **F**–**J** FTs of STEM-ADF images in (**A**–**E**). Reflections forbidden in the cubic structure are highlighted by red circles. (**K**–**O**) enlarged images of the regions marked in (**A**–**E**). Red arrows indicate extra lattice frequency that does not exist in the cubic perovskite structure. STEM-ADF images between 44 and 220 e/Å^2^ are shown and discussed in Supplementary Note 10.

In the case of Cs_0.5_FA_0.5_PbI_3_ (Fig. 4), the first scan (at ~44 e/Å^2^) exhibits the start of a √2 × √2 V^−^_FA_ and V^+^_I_ vacancy superstructure plus weak additional $1/2,-1/2,0$_c_ reflections (along the 〈1–10〉direction) are just evident in the FT (Fig. 4A). These forbidden reflections correspond to an extra lattice frequency in the image in the 〈1–10〉direction (perpendicular to the red arrows). We have carefully examined the first STEM-ADF scans of many Cs_0.5_FA_0.5_PbI_3_ and find that the threshold dose at which such forbidden reflections can be first observed is in the range 44–132 e/Å^2^ (as the first scan is at 44 e/Å^2^, we cannot exclude the possibility that these forbidden reflections might appear below 44 e/Å^2^). We suspect these small variations in the threshold dose for observing these additional $\pm 1/2,\pm 1/2,0$_c_ reflections are related to small composition variations in Cs_0.5_FA_0.5_PbI_3_ (i.e., the Cs^+^/FA^+^ ratio).

In the next scan at 88 e/Å^2^ (Fig S13 B), these $1/2,-1/2,0$_c_ reflections (along the 〈1−10〉 direction) disappear and a new set appears, $-1/2,1/2,0$_c_, along the perpendicular direction, 〈−110〉. By 750 e/Å^2^, the structure appears to have stabilized with all $\pm 1/2,\pm 1/2,0$_c_ reflections present (Fig. 4C). We will call this, Cs_0.5_FA_0.5_PbI_3_—intermediate phase 2. (Again, note that this is not fully stoichiometric due to the loss of A^+^/I^−^.)

Forbidden reflections then gradually disappear with a further increase in total dose, and the structure stabilizes into a square perovskite framework (Fig. 4D, E). By square, we mean a = b and there is a 180° angle between octahedra, in contrast to the octahedral-tilted intermediate phase 2. (We cannot determine the third dimension nor the space group from this single projection.) It is surprising that instead of decomposing quickly into hexagonal PbI_2_, the square perovskite framework remains, even up to a total dose of 3400 e/Å^2^ (Supplementary Note 11). Although the FT of this square perovskite framework structure (Fig. 4E) is similar to that of the pristine cubic perovskite phase, a high density of vacancies (V^−^_FA_, V^+^_I_ and likely V^−^_Cs_) must be present. Moreover, throughout the progression from the 1st to the 2nd intermediate phase and then to the square perovskite framework structure in Cs_0.5_FA_0.5_PbI_3_ (Fig. 4A–D), we notice the formation of local PbI_2_ spherical clusters that fit coherently into the perovskite structure, as well as incoherent Pb clusters which quickly transform into coherent PbI_2_ (Supplementary Note 12).

### Intermediate phases 2—Identification of A-site dependent octahedral tilt phases

**Fig. 5:**
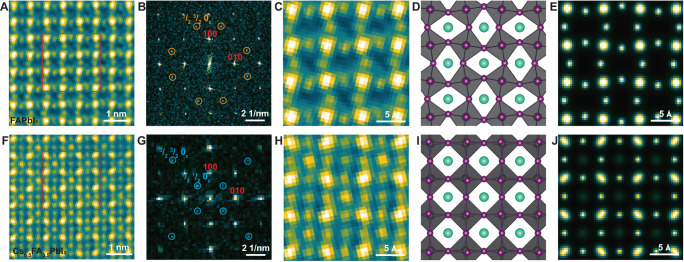
Structural identification of intermediate phase 2 octahedral tilt modes in (A–E) FAPbI_3_ and (F–J) Cs_0.5_FA_0.5_PbI_3_. **A**, **F** STEM-ADF image of fully established intermediate phase 2. **B**–**G** FTs from (**A**, **F**). Circles highlight reflections that are forbidden in the pristine cubic structure. **C**, **H** Unit cell structures of the region marked in (**A**, **F**). **D**, **I** The proposed structures showing different octahedral tilt modes for FAI-deficient FAPbI_3_ (a^0^a^0^c^+^) and Cs_0.5_FA_0.5_PbI_3_ (a^+^a^+^c^0^). Green, grey, and purple atoms represent Cs^+^/FA^+^, Pb^2+^, and I^−^ atomic columns, respectively. **E**, **J** STEM-ADF simulations based on the crystal structures proposed in (**D**, **I**).

The structures of intermediate phase 2 for FAPbI_3_ and Cs_0.5_FA_0.5_PbI_3_ are different, giving rise to different additional reflections as reported above. These two different phase 2 structures are determined here and shown in Fig. 5.

In the case of FAPbI_3_, phase 2 can be attributed to an in-phase octahedral tilt (a^0^a^0^c^+^ in Glazer notation), thus leading to $1/2,3/2,0$_c_ forbidden reflections in the FT (Fig. 5A, B). We further find that this is consistent with a tetragonal $p4/{mbm}$ perovskite structure viewed in the [001] direction (Fig. 5C–E). We note that a similar tetragonal perovskite structure has been observed recently in Cs_0.05_FA_0.78_MA_0.17_Pb(I_0.83_Br_0.17_)_3_ by scanning electron diffraction^41^. The octahedral tilt in that system was proposed by the authors to be intrinsic to the pristine structure and to offer a stabilization mechanism for FA^+^-rich mixed-cation perovskites. Our observations here for FAPbI_3_ are very different. In the case of FAPbI_3_, this octahedral tilt phase is *not* present in the pristine structure. It is unequivocally electron beam induced and occurs at or below 88 e/Å^2^ (at 300 kV in STEM mode).

In the mixed-cation Cs_0.5_FA_0.5_PbI_3_, the ADF-STEM images of phase 2 show an elongation of the intensity maxima at the Pb^2+^/I^−^ column positions. This is found to result from an octahedral tilt mode (a^+^a^+^c^0^ in Glazer notation). (note that we cannot determine the phase ± of octahedral tilts from the projection). Specifically, this image is consistent with the [001] projection of a tetragonal $I4/{mmm}$ perovskite structure (Fig. 5F–J). This specific octahedral tilt mode results in strong forbidden reflections at $1/2,1/2,0$_c_. We further notice that the $p4/{mbm}$ tetragonal phase observed for FAPbI_3_ is also present in local regions of Cs_0.5_FA_0.5_PbI_3_ (Supplementary Note 13). This suggests a local segregation of the A-cations to generate FA^+^-rich regions.

We note it has been reported in some parts of the literature that the pristine structure of Cs_1−*x*_FA_*x*_PbI_3_ is consistent with the *I4/mmm* tetragonal phase^42^, same as in Fig. 5F. Our observations here of the 50/50 Cs/FA, Cs_0.5_FA_0.5_PbI_3_ identify the pristine structure to be the ${pm}\bar{3}m$ cubic phase and the I4/mmm tetragonal phase to be unequivocally electron beam induced, occurring in the range <44 to 132 e/Å^2^ (at 300 kV in STEM mode).

## Discussion

**Fig. 6:**
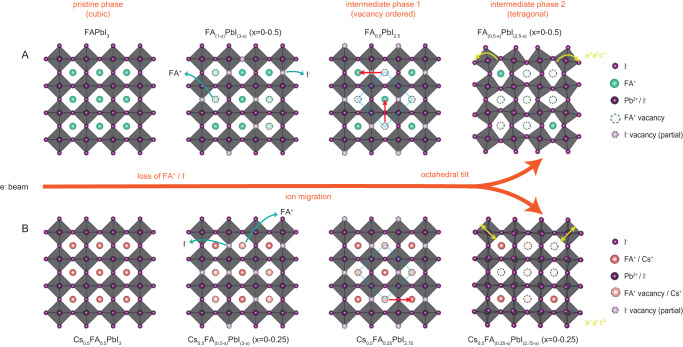
Schematics of the observed ion migration mechanisms and associated phase changes of Cs_1−*x*_FA_*x*_PbI_3_ under the electron beam. **A** FAPbI_3_. It involves an initial loss of FA^+^ and I^−^ (green arrows), followed by the formation of vacancy-ordered superstructure (intermediate phase 1, indicated by blue diamond) through ion migration (red arrows). With the further loss of ions, the pristine cubic phase transforms into a tetragonal phase through octahedral tilt (intermediate phase 2, indicated by yellow arrows). **B** Cs_0.5_FA_0.5_PbI_3_. The loss and migration of ions are thought to be similar to FAPbI_3_, however, the octahedral tilt mode for the intermediate phase 2 is different due to the alloying of Cs^+^ at A-site.

This study provides direct insights, at the atomic-level, into the structural response to stimuli of the mixed cation perovskite Cs_1−*x*_FA_*x*_PbI_3_ and its dependence on A-site composition (Fig. 6). The stimulus used here is an applied electron beam with extremely low current-density. This is used as a proxy with which to study the structural response to light, heat, and electric currents, and to understand at the atomic-level, how these stimuli cause the ion migration and structural degradation that are currently limiting device applications.

Point defects, such as vacancies, in photoactive perovskites are generally believed to be electronically benign, due to the observed long carrier diffusion lengths and low recombination rates^43^. However, while vacancies may be electronically benign, this study shows that they can be structurally toxic, being pivotal to ion migration and structural degradation and thereby undermining the potential of these materials for use in solar cell devices.

Even in the nominally pristine structure, occasional vacancy pairs are evident (Supplementary Fig. 8). Although low density, these provide the initial space essential to permit ion movement and rearrangement. Once a stimulus is applied, additional vacancy pairs can form, further facilitating ion migration and local ordering. These are the first atomic-scale steps to device hysteresis and ultimately structural degradation.

We observe the loss of ion pairs, facilitating subsequent ion migration, unit cell by unit cell, leading sequentially to two intermediate phases, a vacancy-ordered phase, and then A-site dependent octahedral-tilt phases. These insights into the mechanisms of ion loss and migration suggest strategies for reducing ion migration and increasing structural stability which we discuss below.

1. Our first observation is that the structural change commences with a coincident and random loss of cation/anion (FA^+^ and I^−^) pairs, resulting in the formation of vacancies (V^−^_FA_ and V^+^_I_). That is, the loss of an FA^+^ cation will stimulate the loss of an I^−^ anion and vice versa. This applies to both FAPbI_3_ and Cs_0.5_FA_0.5_PbI_3_, however, we observed a slower rate of A-cation loss in the mixed cation compound because the bonding of FA^+^ with PbI_6_ octahedra is weaker than that of Cs^+^. The initial loss of ions would result in a nominal formula of (FA_(1−*x*)_PbI_(3−*x*)_, *x* = 0–0.5) or (Cs_0.5_FA_(0.5−*x*)_PbI_(3−x)_, *x* = 0−0.25). This loss of ions in pairs demonstrates that maintaining local charge neutrality is a dominant driving force within the crystal structure. This suggests that to enhance the structural stability of halide perovskites, it is crucial to suppress vacancies of either type. This in turn suggests that to engineer maximum structural stability of the photoactive phase, it is critical to introduce sources of both cations and ions (such as AX) that can limit or block both vacancy types, both during the initial perovskite material synthesis and for device fabrication purposes. A possible strategy for suppressing vacancy formation is the pinning of A cations and halide sites by enhancing the ionic bonding, for example, through the introduction of B site metal dopants, 2D lattices, or core–shell structures.
2. Our second observation is that in the FA^+^/I^−^ deficient structure with randomly distributed vacancies, FA^+^/I^−^ ions demonstrate high mobility and promptly migrate toward an ordered superstructure. This shows that vacancies play a pivotal role in facilitating ion migration, indeed they may be a necessary condition for ion migration. This further emphasises the need to minimize vacancies in sample preparation, in order to passivate ion migration, improve structural stability and promote superior device performance. We have performed density function theory (DFT) calculations to better understand the A-site cation migration and the role of vacancy in the ion migration. A-site cation migration is not realistic in a perfect perovskite structure with fully occupied ions. DFT results show that the introduction of A-site vacancies will activate the migration of remaining A-site cations (Supplementary Note 14). It suggests that vacancies provide a driving force for the subsequent re-ordering of cations through ion migration. We have also attempted to perform preliminary ab-initio molecular dynamics simulations to model the formation of the superstructure and its diffusion on a larger supercell scale. However, conclusive results have not been reached due to the complexity of the system, as discussed in (Supplementary Note 14).
3. Our third observation is that once vacancies have formed a √2 × √2 superstructure, a relatively stable intermediate phase (with the nominal formula of FA_0.5_PbI_2.5_ or Cs_0.5_FA_0.25_PbI_2.75_), is established in which ion migration is substantially impeded, despite the presence of a significant number of FA^+^/I^−^ vacancies. This suggests the ordered configuration is energetically favourable, requiring a high activation energy to move an ion pair and break the local symmetry. It demonstrates how a well-ordered structure can discourage ion migration and suggests the design of well-ordered crystal structures, even those with ordered vacancies, might be one avenue for mitigating ion migration. Furthermore, it also indicates a potential atomic-level mechanism for how non-reversible ion migration and hysteresis might occur, namely, by driving ions into an ordered, energetically favourable superlattice from which larger energy is required to subsequently move them.
4. Our fourth observation is that, with the further loss of ions, the pristine perovskite Pb-I framework can no longer be maintained, and a second intermediate phase is formed through octahedral tilt resulting in a tetragonal phase transition. At this stage the nominal formula corresponds to (FA_(0.5−*x*)_PbI_(2.5−*x*)_, *x* = 0–0.5) or (Cs_0.5_FA_(0.25)_PbI_(2.75−*x*)_, *x* = 0−0.25) before completely decomposing into PbI_2_ (Supplementary Fig. 17). The octahedral tilt mode (or symmetry of the tetragonal perovskite phase) depends upon the type of A-site cation. The observed correlations between A-site cations (type and occupancy) and the Pb–I framework highlight how the A-site can influence structural stability by tuning the octahedral tilt mode.

In summary, these observations reveal at the atomic scale the mechanisms by which ion migration occurs. This in turn suggests several strategies for designing the structure of perovskite photoabsorbers that will inhibit ion migration and promote structural stability, as follows:

- We find ion migration requires vacancies and these occur as cation/ion pairs. Chemical processes that block the formation of either vacancy type (cation or ion), will inhibit ion migration and enhance structural stability. Chemical processes and/or composition engineering that block both types (cation and ion), may block ion migration altogether.
- Well-ordered vacancy superstructures have higher stability and might be incorporated deliberately into the structure to discourage ion migration (for example, through low-temperature annealing in chemical processing).
- Structural stability can be enhanced through octahedral tilting, which might be induced through A-site composition engineering.

Since other stimuli such as high-intensity light, heat, or electrical fields may induce vacancy formation and ion migration in an analogous manner, these findings may provide fundamental instructions to guide the development of stable optoelectronic devices under various conditions.

## Methods

### Chemicals

All chemicals were purchased from Sigma Aldrich and used without purification unless otherwise noted. Caesium carbonate (Cs_2_CO_3_, 99.9%) lead iodide (PbI_2_ 99.9985%, Alfa Aesar), oleic acid (OA, technical grade 90%), oleylamine (OLA, technical grade 70%), 1-octadecene (ODE, technical grade 90%), toluene (anhydrous 99.8%), hexane (anhydrous, 95%), methyl acetate (MeOAc, anhydrous 99.5%), ethyl acetate (EtOAc, anhydrous 99.5%), lead(II) acetate trihydrate (Pb(OAc)_2_·3H_2_O, 99.999%), formamidine acetic acid salt (FA-acetate, ≥99%), oleylammonium iodide (OLA-I, ≥99%, Xi’an Polymer Light Technology Corp).

### Synthesis of CsPbI_3_ QDs

Cs-oleate was obtained by dissolving 0.1 g of Cs_2_CO_3_ into 0.4 ml of OA and 10 ml of ODE, and the mixture was loaded into a 50-ml three-neck flask and stirred under vacuum for 30 min at 120 °C. After fully dissolving, the Cs-oleate in ODE was stored under nitrogen until it was used. PbI_2_ (0.4 g), ODE (20 ml), OA (2 ml), and OLA (2 ml) were stirred in a 100-ml flask and degassed under vacuum at 120 °C for 1 h. The flask was then filled with N_2_ and kept under constant N_2_ flow. The temperature was increased to 170 °C, and then 3.4 ml of the Cs-oleate precursor was swiftly injected into the mixture. After 10 s, the reaction was quenched by immediate immersion of the flask into an ice bath. After cooling to room temperature, 30 ml of MeOAc was added, and the mixture was centrifuged at 6440 xg for 10 min. The resulting QD precipitate was dispersed well in 2 ml of hexane and was centrifuged again at 906×*g* for 2 min to remove agglomerations. The concentration of the obtained QDs ink was further adjusted to 50 mg ml^−1^ by adding the proper amount of hexane. Then the CsPbI_3_ QDs ink was stored under nitrogen until use.

### Synthesis of FAPbI_3_ QDs

Pb(acetate)_2_·3H_2_O (0.152 g), FA-acetate (0.157 g), ODE (16 ml) and OA (4 ml) were added in a 100-ml three-neck flask and dried under vacuum for 30 min at 40 °C. The mixture was then heated to 80 °C under an N_2_ atmosphere, followed by an injection of OLA-I (0.474 g dissolved in 4 ml of toluene). After 30 s, the reaction mixture was cooled in the water bath. After cooling to room temperature, 20 ml of MeOAc was added, and the mixture was centrifuged at 6440×*g* for 5 min. The resulting QD precipitate was dispersed in hexane and was centrifuged again at 1610×*g* for 4 min to remove agglomerations and impurities. The concentration of the purified QDs ink was further adjusted to 50 mg ml^−1^ by adding a proper amount of hexane. Then the QDs ink was stored under nitrogen until use.

### Synthesis of Cs_0.5_FA_0.5_PbI_3_ QDs

Cs_0.5_FA_0.5_PbI_3_ QDs were obtained by cation-exchange reactions: The stored CsPbI_3_ QDs and FAPbI_3_ QDs were mixed under N_2_ atmosphere with a calculated volume ratio to guarantee the desired composition of the QDs. The ligand-assisted cation-exchange reaction was completed in 60 min at room temperature. The obtained QD ink was kept in an N_2_-filled glovebox for an additional 12 h to guarantee the even distribution of surface ligands.

### Ligand density reduction

The surface ligand density of the obtained FAPbI_3_ QDs and Cs_0.5_FA_0.5_PbI_3_ QDs were further reduced by adding EtOAc (volume ratio of QD solution to EtOAc was 1:1) into the QD inks and centrifuged at 6440×*g* for 5 min. The resulting QD precipitate was dispersed in hexane and was centrifuged again at 1610×*g* for 4 min to remove agglomerations. The concentration of the QD inks was further adjusted to 50 mg ml^−1^ by adding a proper amount of hexane. The purification process, including the mixing of QD inks with MeOAc or EtOAc, the centrifuge, and the dispersion of QDs in hexane was conducted in dry air with a relative humidity between 20% and 25%.

### TEM specimen preparation

QD solutions were stored in the glove box filled with dry N_2_. Ultrathin carbon-coated Cu TEM grids were plasma-cleaned under H_2_/O_2_ for 30 s before use. A sample of QD solution was taken and immediately dropped onto the TEM grid and allowed to dry for 30 s. This whole sample preparation was conducted in the glove box (N_2_). The prepared TEM specimen was transferred from the glove box to the TEM room, using a homemade stainless steel vacuum transfer unit. The total time of TEM specimen exposure in the atmosphere is <30 s.

### TEM characterization

STEM-ADF was carried out using an FEI Titan^3^ 80-300 FEG-TEM equipped with probe and imaging spherical aberration correctors. All images were acquired at 300 kV, a 15 mrad probe-forming aperture, and 39–200 mrad detector collection angle. Electron dose was measured using an electron microscope pixel array detector (EMPAD) based on a measurement of 10,000 frames of the vacuum probe. HR-TEM was performed using a Thermo Fisher Scientific Spectra φ FEGTEM equipped with a monochromator and probe and imaging C5 aberration correctors. HR-TEM images were acquired on a Gatan K3 camera in counting mode at 75 fps. All TEM experiments followed a strict “shoot blind” protocol whereby a fresh region of the specimen is first exposed to the electron beam at the start of data acquisition and only exposed for the duration of data acquisition. In particular, no electron dose was applied to the material for tilting to a zone axis or adjusting imaging parameters. Images were post-filtered with a combined Bragg filter and a Butterworth filter and corresponding raw images are given in the supplementary material.

### STEM simulations

STEM-ADF images were simulated using a GPU-enhanced frozen-phonon multislice code (µSTEM). The simulations employed supercells by tiling perovskite unit cells by 8 × 8 (supercells in size 5 × 5–10 × 10 nm), combined with 1024 × 1024 pixels to ensure accuracy. Experimental conditions were used as the parameters for the calculation of the STEM images. 30 frozen phonon passes were calculated.

### DFT calculations

DFT as implemented in the Vienna Ab Initio Package (VASP)^44^ is used to study the Cs_*x*_FA_1−*x*_PbI_3_ perovskite structure. The GGA-PB^45^ functional is considered for all the calculations. To minimize the effect of periodic boundary conditions on atomic interactions, a supercell consisting of 8 unit cells with 96 atoms (12.703 Å × 12.703 Å × 12.703 Å) for FAPbI_3_ and a supercell with 40 atoms (12.564 Å × 12.564 Å × 12.564 Å) for CsPbI_3_ are considered for this study. A *k*-point of 1 × 1 × 1 at the Γ-point is applied for all the calculations. To compare the migration energy barrier (NEB) of FA and Cs ions in the structure of FAPbI_3_ and CsPbI_3_, nudge elastic band calculations with a force tolerance of 0.03 eV Å^−1^ and an energy cutoff of 520 eV are carried out. To make the initial and final structures, one FA and I and similarly one Cs and I are removed from FAPbI_3_ and CsPbI_3_ structures (FA_0.875_PbI_2.875_ and Cs_0.875_PbI_2.875_) and DFT ground state optimization is used to converge the initial and final migration points. The energy-optimized structures are used to generate six intimidating images for NEB calculations.

### Ab-initio molecular dynamics (MD) simulation

The samples that were used for NEB calculations are also being used for Born–Oppenheimer molecular dynamics simulations. Ab initio MD simulations were performed using the CP2K/Quickstep package^46^. The Perdew–Burke–Ernzerhof (PBE) generalized gradient approximation (GGA)^45^ was selected for the DFT exchange-correlation functional. To correct for van der Waals interactions the DFT-D3^47^ method was used. Pseudopotentials of Goedecker, Teter, and Hutter (GTH)^48^ were employed and the DZVP-MOLOPT-SR-GTH^49^ was selected as basis set. This is a Gaussian and plane-wave (GPW)^50^ basis and a cutoff energy of 280 Ry was selected. A 1 × 1 × 1 *k*-point mesh (Γ point) was used in all calculations. For ab initio MD simulations, the equations of motions were integrated using a velocity Verlet algorithm with a time step of 1 fs.

### Supplementary information

Supplementary Information

Peer Review File

### Source data

Source Data

## Data Availability

All relevant data generated in this study are provided in the paper and its Supplementary Information files. Source data has also been deposited in Figshare under the accession link 10.6084/m9.figshare.24615702^51^. Source data are provided with this paper.
