# Supplementary material for: The role of ion migration, octahedral tilt, and the A-site cation on the instability of Cs1-xFAxPbI3
Source: Nat Commun. 2023 Dec 22;14:8523. doi: 10.1038/s41467-023-44235-6 (PMC10739958; doi:10.1038/s41467-023-44235-6)
Supplement: Supplementary file 1 — Supplementary Information [file 41467_2023_44235_MOESM1_ESM.pdf]

Supplementary Information

The role of ion migration, octahedral tilt, and the A-site cation on the instability of $Cs_{1-x}FA_xPbI_3$

Weilun Li[1*], Mengmeng Hao[2], Ardeshir Baktash[3], Lianzhou Wang[2,3*], Joanne Etheridge[1,4,5*]

*e-mail:

weilun.li@monash.edu

l.wang@uq.edu.au

joanne.etheridge@monash.edu

This PDF file includes:

Supplementary Note 1 to 14

Supplementary Figures 1 to 19

Appendix

Supplementary References

Supplementary Note 1

Quantification of total electron dose in STEM mode

A precise measurement of the total electron dose applied to a region of interest is essential, if we are to make meaningful TEM measurements of beam-sensitive materials, such as hybrid perovskites. The total electron dose is the number of electrons per second per area (current density) summed over the total time the area is illuminated. Probe current in a TEM can be measured by different methods. The probe current that is used for atomic resolution imaging in STEM of hybrid perovskites is generally much less than 5 pA. In this dose range, measurements made by a direct electron detection camera offers the most precise approach for measuring current, compared with alternative methods such as using a built-in screen amperemeter, Faraday cup or drift tube[1].

In this work, a fast direct detection camera was used – an EMPAD from Thermo Fisher Scientific[2]. Under the same electron-optical imaging conditions used in this work for STEM-ADF imaging, the probe was moved to the vacuum (called “vacuum probe”) and was recorded in the diffraction plane by the EMPAD running at 1000 fps. 10,000 frames of the vacuum probe were collected in a 10 s exposure time and averaged. In this way, all electrons in the incident probe were collected. The intensity unit was then converted to the number of electrons, using the antilog-digital-unit (ADU) determined at 300 kV. Knowing the acquisition time of a single frame (1 ms), the probe current was measured to be 1.48 pA.

To obtain the total dose in STEM mode, we can multiply by the probe dwell time (2 µs) and divide by the exposure area (pixel size = 0.647 Å), to give an estimate of the total dose (44.194 e/Å^2).

Quantification of total electron dose in TEM mode

HR-TEM images were collected by a direct detection electron camera (K3-iS from Gatan Inc) in a counting mode. In this mode, the material in the field of view was illuminated uniformly by a broad, parallel electron beam. The total number of electrons hitting the camera was read directly and the total dose of a single frame in (e/Å^2) was calculated, with extremely high precision.

Supplementary Note 2

Observation of iodine vacancy √2 x √2 superlattice

The ordered high-low-high image intensities at the Pb^{2+}/I^- and I^- column positions observed in STEM-ADF images suggest that there is a corresponding √2 x √2 superstructure of iodine vacancies, V^+_I. However, this interpretation needs to be checked via image simulations. In particular, the absence of FA^+ cations at the A-site might influence the image intensity associated with neighbouring columns via dynamical scattering[3,4]. In the simulated STEM-ADF image containing only ordered $V^-_{FA,}$ and no iodine vacancies the intensity oscillations at the Pb^{2+}/I^- and I^- column positions are not observed, (Supplementary Figure 1(A, B)), inconsistent with the experimental data. In addition, as shown in the crystal model in Supplementary Figure 1C, geometrically, when a vacancy is present at the A-site, the four neighbouring I^- columns or Pb^{2+}/I^- columns are symmetry equivalent, due to the cubic symmetry of its unit cell (space group = $pm\overline{3}m$). This means that in the STEM-ADF image, any influence of the central FA vacancies on the neighbouring sites would be equivalent and cannot lead to the experimentally observed ordering pattern of Pb^{2+}/I^- and I^- column intensities. We have similarly performed the simulations with ordered I vacancies (Supplementary Figure 1(D-F)), which shows that ordered I vacancies also cannot lead to the ordered pattern at the A-site.

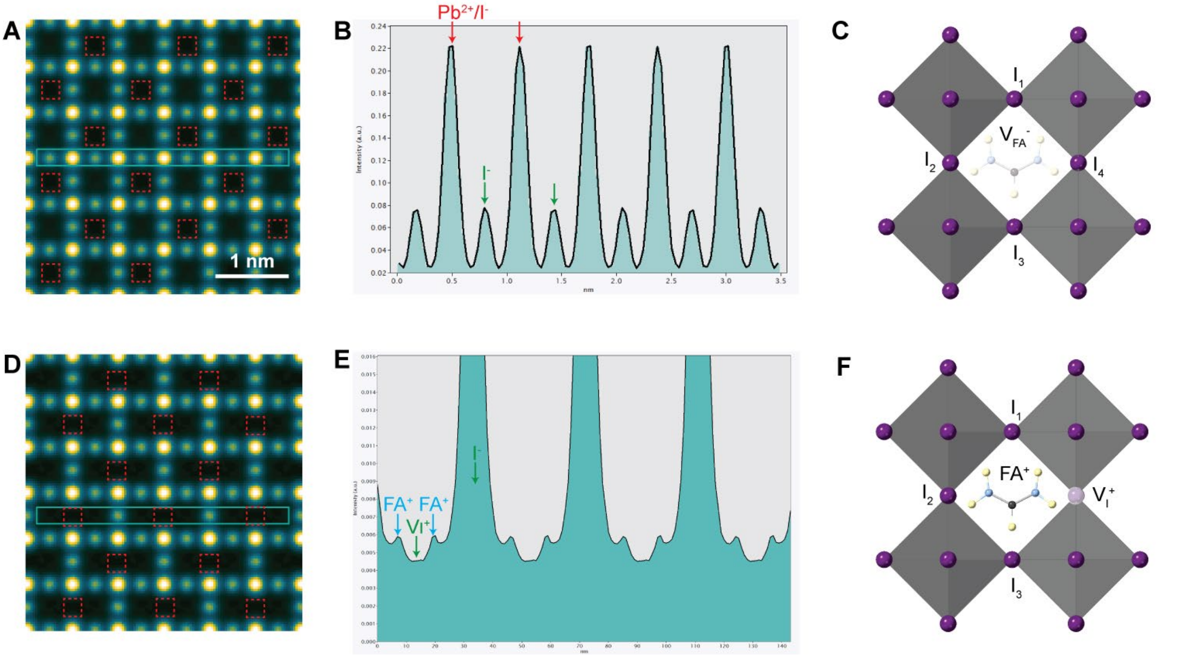

Supplementary Figure 1. STEM-ADF simulations of cubic $FAPbI_3$ with ordered vacancies. (A) simulated STEM-ADF under the same imaging conditions as that in Fig. 1. (B) Intensity line profile of the region marked in (A). (C) Crystal model of cubic perovskite structure containing V^-_{FA}. (D-E) STEM-ADF simulations of the structure with ordered I^- vacancies (F). Dashed squares indicate vacancies in (A, D). Shadowed atoms in (C, F) indicates the position of vacancies.

Supplementary Note 3

Comment regarding determining the dose threshold from the Fourier transform of atomic resolution (S)TEM images

We would like to make an important point here regarding the identification of the electron dose at which damage occurs. This is often done through the identification of additional reflections in the Fourier transform (FT) of an image that are inconsistent with the pristine structure. We emphasise here that subtle, local damage can occur on a scale that is too small and aperiodic to be detectable in the FT of the image, hence FTs may fail to detect the early stages of damage and are not a reliable measure of the lowest dose limit at which electron damage might occur. The current work is a case in point, which we highlight in this supplementary note.

It is evident that the structure imaged in Supplementary Figure 2A (unfiltered raw image of Fig. 1E) is not consistent with the established cubic structure of $FAPbI_3$, despite the use of the lowest achievable dose conditions for the STEM-ADF image. STEM-ADF images are composition-sensitive so the existence of A-site vacancies can be observed directly from the image as indicated by the lower image intensity within the dashed red squares. Furthermore, where they occur, these vacancies tend to be ordered, as seen from the image and corresponding intensity line profile in Supplementary Figure 2C.

The observed vacancy superstructure has a lower symmetry presumed to have been induced by exposure to the electron beam. However, the image intensity modulations generated by this superstructure are too subtle and too localised to be detectable as additional reflections in the corresponding FT of the image, which instead appears to be consistent with the pristine cubic structure (Supplementary Figure 3A). To expand on this point, the signal-to-noise ratio (SNR) of the first STEM-ADF image (Supplementary Figure 2A) is extremely low (because it was deliberately taken at the lowest possible STEM-ADF electron dose conditions). In addition, due to the low electron dose used in this initial image, minimal damage has been done, so the superstructure is only present in a few local regions, insufficient to generate additional ‘narrow-frequency-band’ reflections above the broad-band noise in the FT.

The electron dose at which additional reflections appear in the FT of TEM images has been commonly used to estimate the dose threshold at which structural change is induced by the electron beam[5]. The observations here are a reminder that important structural changes (such as loss of ions and reordering) can occur *well before* they are detectable with in the image FT. These changes can initially be too subtle to be detectable as additional (‘forbidden’) reflections in image FTs or potentially in SAED patterns. Hence the electron dose required for damage may be overestimated from image FTs. In the present case of $FAPbI_3$, it is evident that an ordered vacancy superlattice can be induced with just 44 $e/Å^2$.

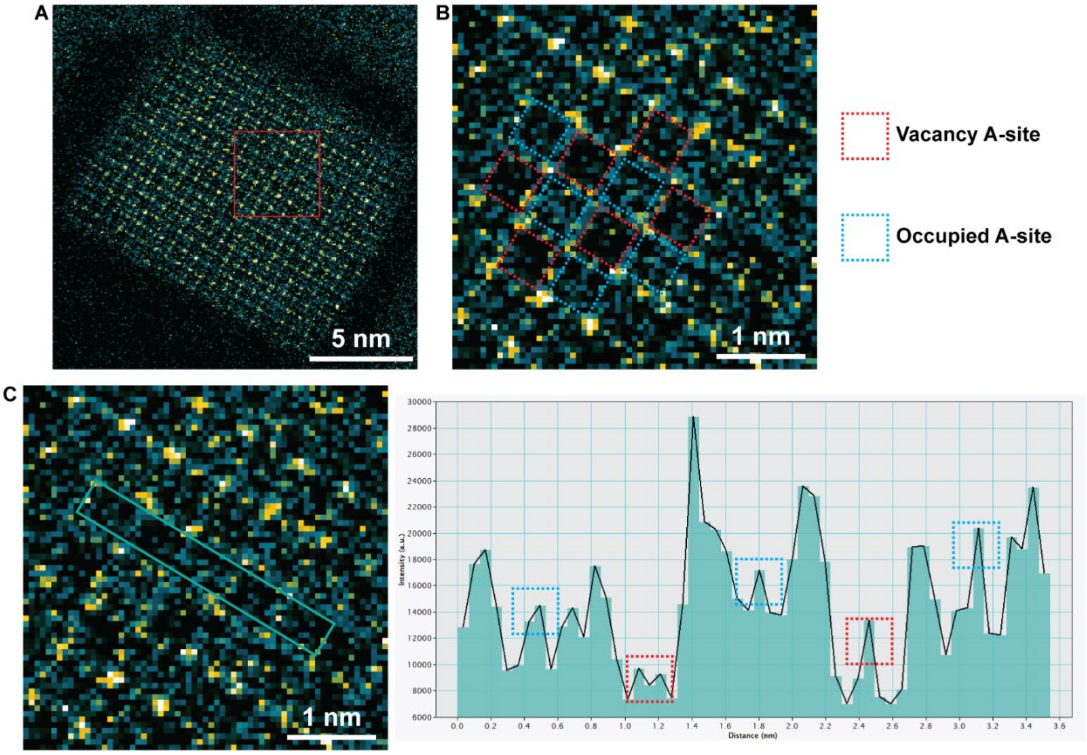

Supplementary Figure 2. $Cs_{0.5}FA_{0.5}PbI_3$ showing, localised vacancies detectable in the raw data image, presumed to be due to initial beam damage. (A) Raw STEM-ADF image of $Cs_{0.5}FA_{0.5}PbI_3$ taken at 44 e/Å^2. (B) Enlarged image of region marked in (A). (C) Intensity line profile measured from the green rectangle. Positions in the plot corresponding to the A-site atomic positions are highlighted by squares (blue for higher intensity, red for lower intensity and hence lower occupancy).

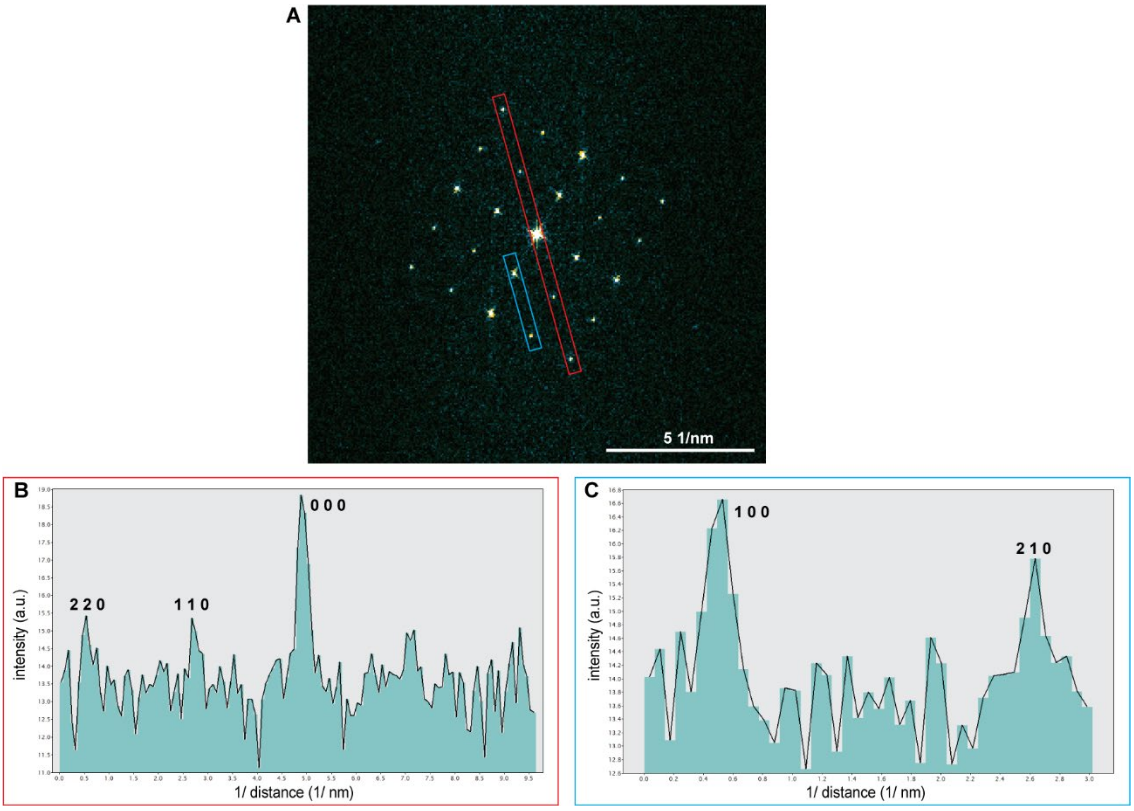

Supplementary Figure 3. $Cs_{0.5}FA_{0.5}PbI_3$ - FT of lowest dose, raw STEM-ADF image (Supplementary Figure 2) - the signal from the local vacancies is not detectable above the noise in the FT. (A) FT of the STEM-ADF in Supplementary Figure 2A. (B)

Intensity line profile from the region highlighted by red shows no additional $^{1}/_{2}, ^{1}/_{2}, 0_c$ reflections that would be associated with the ordered vacancies. (C) Intensity line profile from the region highlighted by blue shows no additional $^{3}/_{2}, ^{1}/_{2}, 0_c$ reflections that would be associated with the ordered vacancies.

Supplementary Note 4

Post filtering of low dose STEM-ADF images

Superstructures formed in the material should lead to extra reflections in the corresponding diffraction patterns. In the cubic perovskite structure here, the √2 x √2 ordering of V^{-}_{FA} and V^{+}_{I} will result in extra reflections at the centre positions among each primary reflections of the cubic structure, for example $^{1}/_{2}, ^{3}/_{2}, 0_{c}$ and $^{1}/_{2}, ^{3}/_{2}, 0_{c}$. However, these extra reflections arising from superstructures can be much weaker in intensity than the primary reflections, making them particularly hard to detect. This is particularly true for the case of Fourier transforms of low-dose STEM-ADF images, where several different sources of noise contribute strongly to the resulting images, such as shot noise.

To reduce the effect of noise, we applied different filters, all of which enhance the contrast from the √2 x √2 superstructures in the image (Supplementary Figure 4(B-D)). It is important that any filtered images should be compared with raw images to make sure that post-filtering does not create features/artefacts that are not present in the original raw images. In Supplementary Figure 5, the filtered images are compared with the raw image. The combination of Bragg-Butterworth filter in Supplementary Figure 5B shows A-site vacancies and its position and ordering are the same as revealed in the raw image in Supplementary Figure 5A (same figure as Supplementary Figure 2A). Furthermore, the filtering helps to visualise the halide columns. The Bragg-Butterworth filter has also been utilised in the literature for the analysis of low-dose STEM-ADF images of $FAPbI_3$, and it has not been found there to show structural artefacts. All structures (e.g. vacancies and ordering) were also evident in the raw images[6].

We also applied other commonly used image filters, such as the ABSF filter and the Winer filter to the low-dose STEM-ADF images (Supplementary Figure 5A), see Supplementary Figure 5(C, D). They also show the same A-site vacancies and ordering that exist in the raw image.

The only Fourier transform to show additional reflections associated with the vacancy ordering (which is unequivocally evident in all the images) is the FT of the Bragg-Butterworth filter is the most efficient image (Supplementary Figure 4B). This is to be expected, as the Bragg-Butterworth filter is the most efficient in terms of the balance between de-noising and the retention of high frequency structural information.

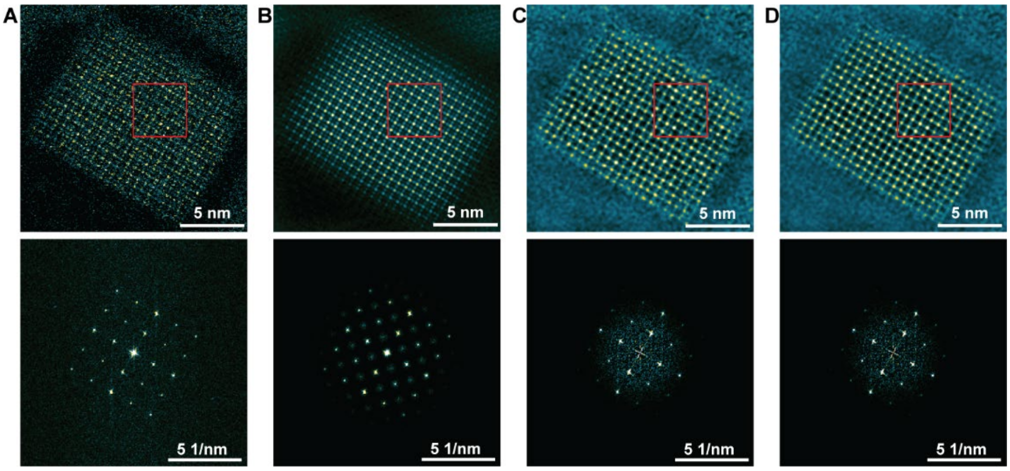

Supplementary Figure 4. Post-filtering of an example lowest dose STEM-ADF image taken from $Cs_{0.5}FA_{0.5}PbI_3$, with a total dose at 44 e/Å^2. (A) Raw STEM-ADF image. (B) Image filtered by a combination of Bragg filter and Butterworth filter. (C) Image filtered by a Winer filter. (D) Image filtered by an average background subtraction filter (ABSF). Corresponding FTs are given below the overview images.

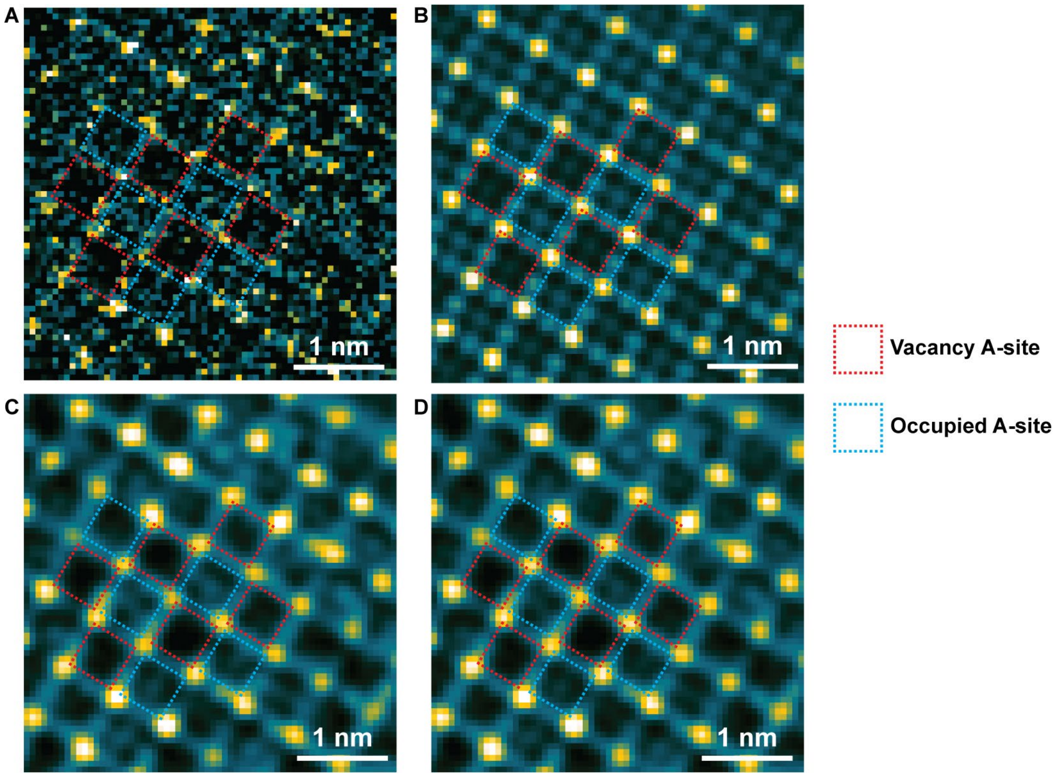

Supplementary Figure 5. Post-filtering of an example lowest dose STEM-ADF image taken from $Cs_{0.5}FA_{0.5}PbI_3$, with a total dose at 44 e/Å^2. Enlarged images of marked regions in Supplementary Figure 4. (A) Raw image, (B) Bragg-Butterworth filtered image, (C) Winer filtered image, (D) ABSF filtered image. All the filtered images exhibit vacancies and ordering in the same location as the original, raw unfiltered image.

Supplementary Note 5

Quantitative estimation of A-site vacancy and A-site/halide vacancy pairs

A more quantitative analysis of the A-site column intensity in Fig. 1 is shown in Supplementary Figure 6, where the intensity in a Voronoi cell around each atomic column position has been integrated. This enhances the intensity variation evident at the A-site columns, with a lower intensity at A-site columns attributed to vacancies in that column. The ordering of A-site vacancies results in a checkered pattern in the integrated A-site intensity map. In this initial image, the checkered pattern is only observed in some areas, which is likely because, at this low dose, there is limited damage so relatively few A-site vacancies have been generated.

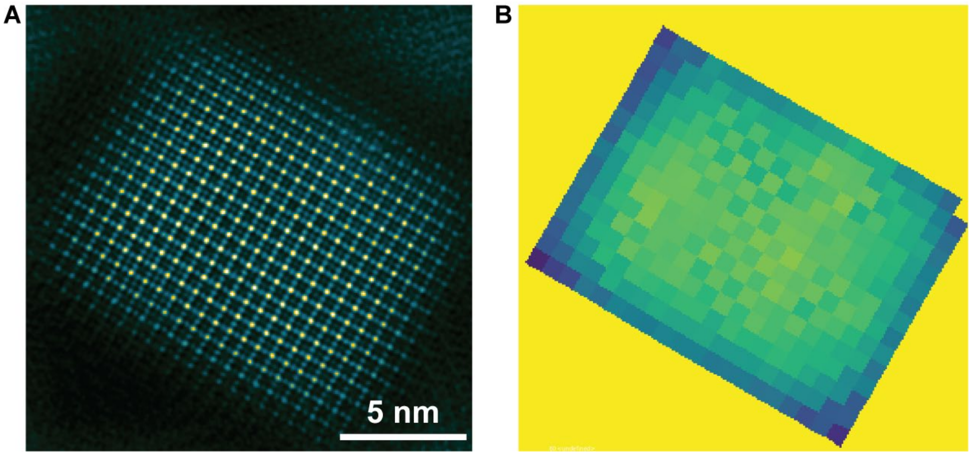

Supplementary Figure 6. Quantitative analyses of A-site intensity from low-dose STEM-ADF image of $Cs_{0.5}FA_{0.5}PbI_3$. (A) Filtered image (the same as that in Fig. 1E), (B) map of integrated intensity of A-site columns.

As discussed in the main manuscript. Vacancy and vacancy-ordered superstructures were observed for both A-sites and halide sites (Fig 1). The position of vacancy containing columns is highlighted in Supplementary Figure 7. This suggests that cation vacancies and anion vacancies occur in pairs and form correlated superstructures. The number of cation and anion vacancies are expected to be identical to ensure charge neutrality and this appears to be the case from the image, in so far as it can be quantified.

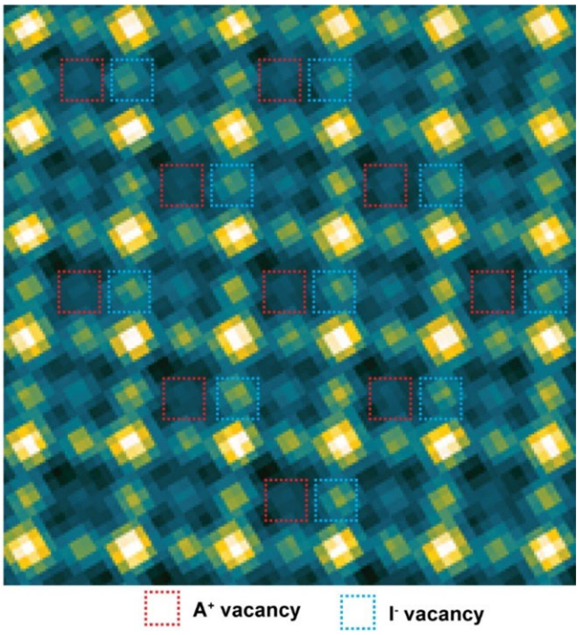

Supplementary Figure 7. Analyses of A-site vacancies and I-site vacancies from low-dose STEM-ADF image of $Cs_{0.5}FA_{0.5}PbI_3$ (the same as Fig. 1F).

Supplementary Note 6

HR-TEM image of $FAPbI_3$ at a total dose of 7.8 e/Å^2

By summing the first 6 HR-TEM images, each taken with ~ 1.5 e/Å^2, the atomic structure of the material can just be resolved and is sufficient to do quantitative measurements (Supplementary Figure 8). Although the precision of the column intensity analysis is affected by shot noise at such low dose, it still shows intensity variations and even ordered structures of V^{-}_{FA} and V^{+}_{I} in local regions. It is possible that these vacancies are intrinsic to the unexposed, pristine $FAPbI_3$, for example, if prepared under the conditions of insufficient surface ligand (oleic acid). It could also be possible that they were caused by the electron beam, even at this extremely low dose. It is not possible from this data to know whether one or both explanations are correct. Non-local averaging of unit cells can enhance the SNR of HR-TEM images, which can improve the atomic-resolution HR-TEM image at a dose < 1 e/Å^2 [7]. However, applying a non-local averaging method to an image of random vacancies might result in the loss of structural features intrinsic to the pristine material and misinterpretation of the image, so this was not used here.

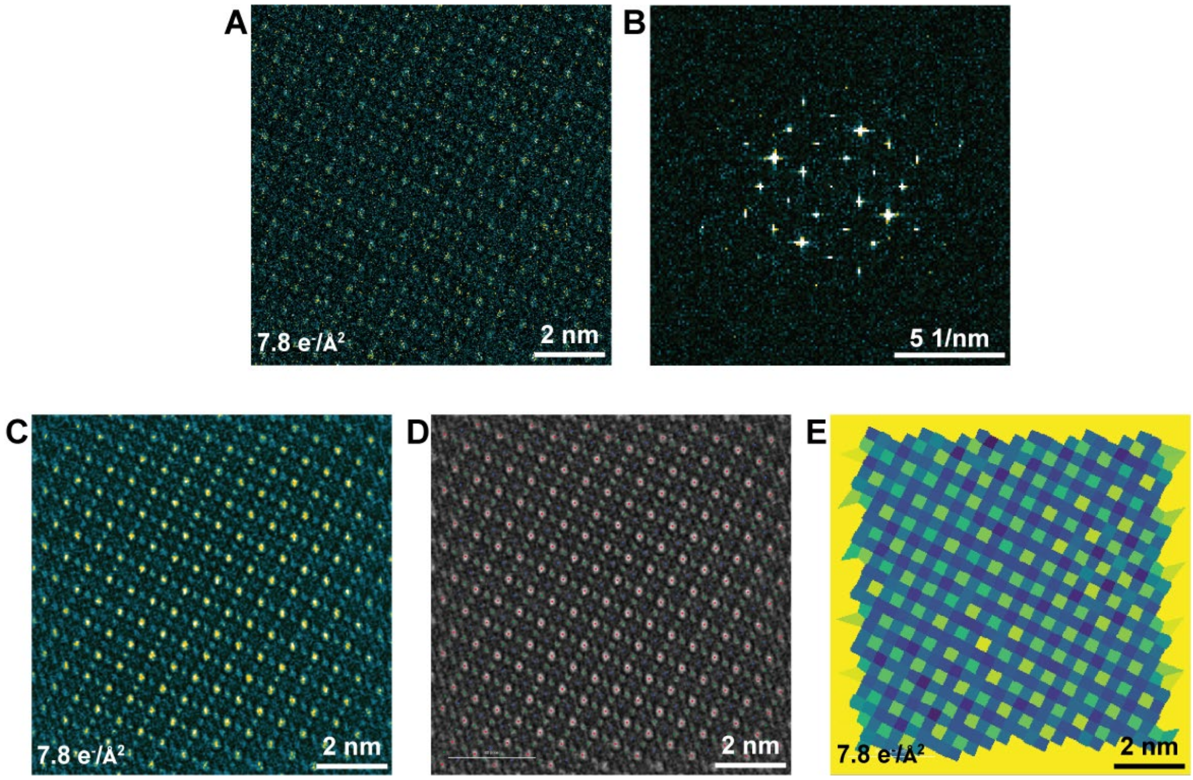

Supplementary Figure 8. Extreme low dose HR-TEM images of $FAPbI_3$. (A) Raw HR-TEM image at 7.8 e/Å^2. (B) FT of (A). (C) Filtered image of (A). (D) Fitting of atomic column positions. (E) integrated column intensity map.

Supplementary Note 7

Determination of atom types in phase-contrast HR-TEM images

Unlike STEM-HAADF images where the intensity of atomic columns can be related directly to the type and number of atoms in the column, contrast in phase-contrast HR-TEM images can be significantly affected by specimen thickness and imaging conditions, such as defocus and lens aberrations. In the HR-TEM images in Supplementary Figure 9, three levels of intensity, A to C, are observed at atomic column positions. We identify the atom types associated with these 3 intensity levels and positions, as follows:

Firstly, the intensity B can be determined as corresponding to the corners of the octahedra due to their unique arrangement relative to A and C. The corners contain iodine only, so we allocate the B-intensity to pure iodine columns. There are two observations that indicate that the C-type column corresponds to the FA^+ and the A-type column correspond to the Pb^{2+}/I^-, as follows:

(1) Column shape. The projection shape of Pb^{2+}/I^- columns in this projection is expected to be spherical while FA^+ molecule is non-spherical and is also expected to rotate in arbitrary orientations at this temperature (Supplementary Figure 9C). In the HR-TEM image, A-type intensity distribution is consistently observed to have a spherical shape consistent with Pb^{2+}/I^- (or a slight elliptical shape due to a slight tilt away from the zone axis), however the C-type intensity distribution is much more variable in shape consistent with FA^+.

(2) Vacancy. From the STEM-ADF image (Fig. 1), where the atom types can be easily determined, we observed the occupancy of the FA^+ columns vary significantly, while the occupancy of the Pb^{2+}/I^- columns is much more stable (at least at the low doses used for this image). In the HR-TEM image, A-type intensity shows a minor intensity variation which the C-type intensity shows significant variations. This is again consistent with the A position corresponding to Pb^{2+}/I^- columns and C corresponding to FA^+ columns.

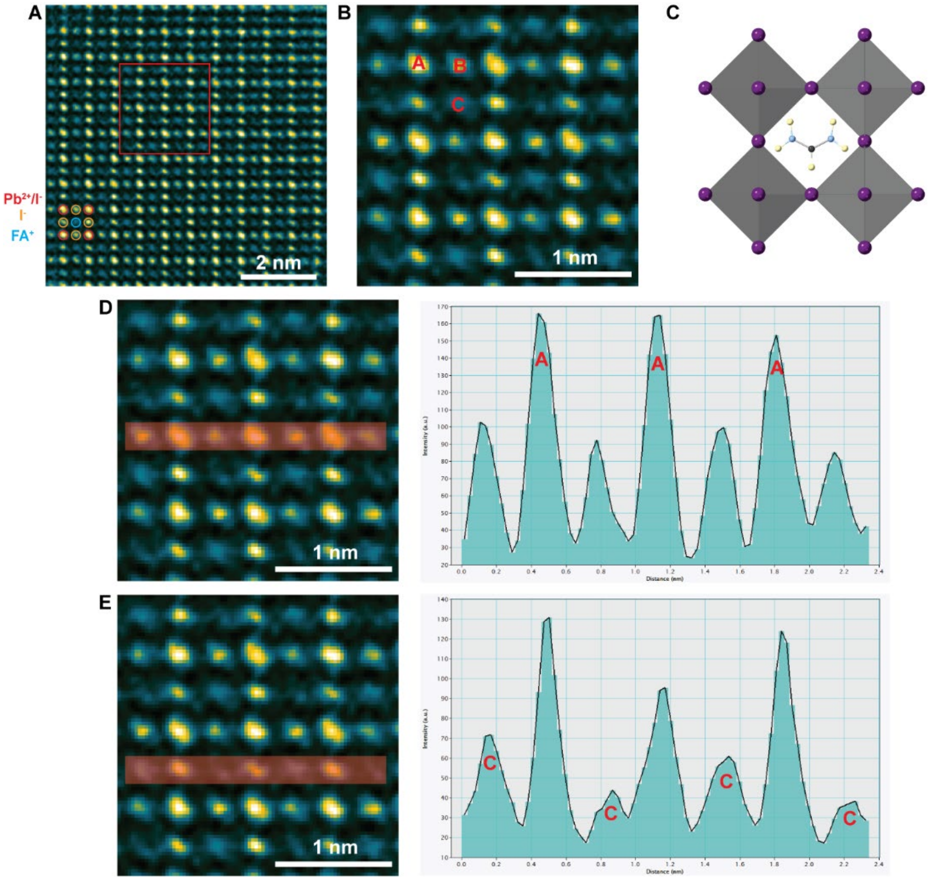

Supplementary Figure 9. Phase-contrast HR-TEM image of $FAPbI_3$ – identification of atom types. (A) Same image as Fig. 2D at 245 e/Å^2. (B) Enlarged image of the region marked in (A). (C) structure model of $FAPbI_3$ unit cell with FA^+ perpendicular to the view direction. (D) Intensity line profile across A-type intensity maxima. (E) Intensity line profile across C-type intensity maxima.

Supplementary Note 8

Loss and ordering of I^- vacancies

The loss and reordering of FA^+ were found to be complemented by the loss and reordering of I^- anions in both Pb^{2+}/I^- and I^- columns. The loss of I^- and reordering of V^+_I is evident in the intensity line profiles in Supplementary Figure 10. Initially, intensity variations are shown for both I^- containing columns, I^- and Pb^{2+}/I^- columns (Supplementary Figure 10A). Then ordered (high-low-high) intensity oscillations gradually become clear (Supplementary Figure 10(B-D)).

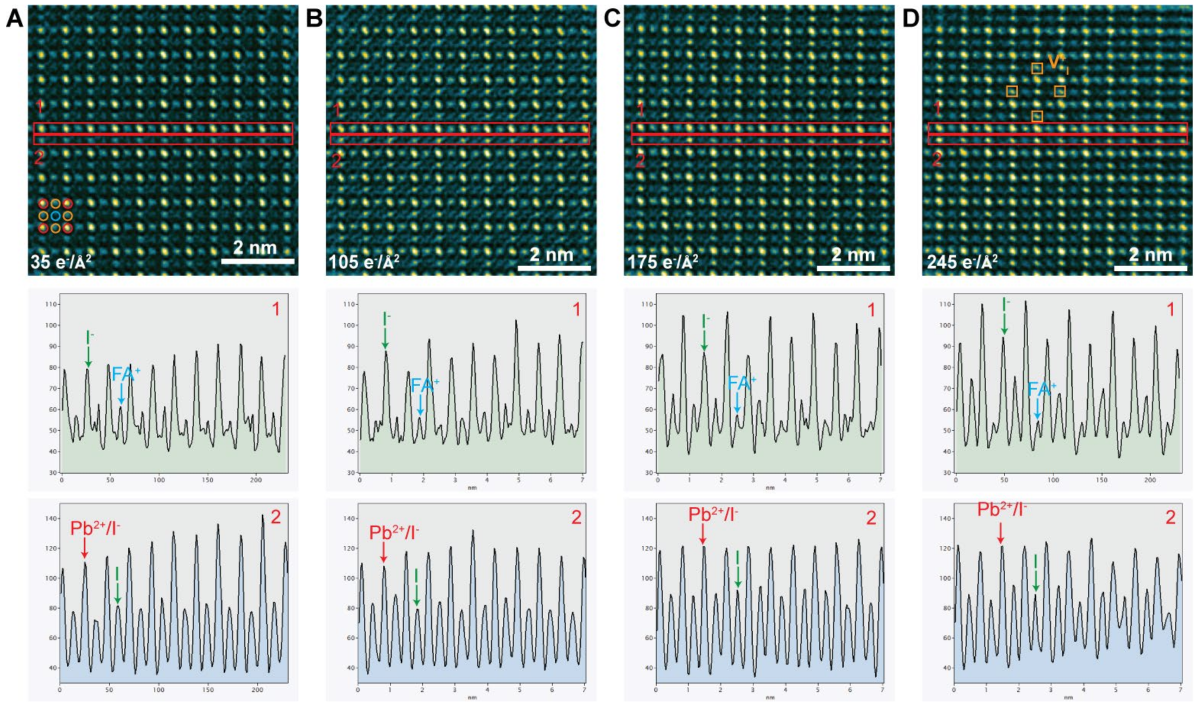

Supplementary Figure 10. Atomic-scale HR-TEM images revealing initial loss of I^- and subsequent unit cell by cell I^- ion migration. (A) 35 e/Å². (B) 105 e/Å². (C) 175 e/Å². (D) 245 e/Å². Intensity line profiles are extracted from region 1 and region 2, respectively.

The migration of I^- ions via vacancies V^+_I is shown in the integrated column image intensity maps in Supplementary Figure 11. The marked Pb^{2+}/I^- columns positions have higher intensities (occupied sites) in Supplementary Figure 11(A, B); however, they become lower intensity (vacancy sites) later in Supplementary Figure 11(C, D), as I^- ions migrate to form an ordered √2x√2 pattern of I^- ions and V^+_I, complementing that of the FA^+ cations.

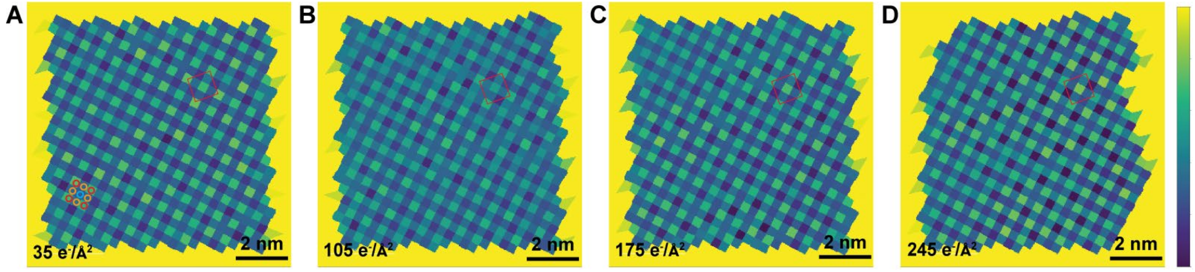

Supplementary Figure 11. integrated column intensity maps. (A) 35 e/Å². (B) 105 e/Å². (C) 175 e/Å². (D) 245 e/Å². Red square corners indicate four Pb^{2+}/I^- columns at its corners.

Supplementary Note 9

Effect of tilt and other parameters on the detection of V^{-}_{FA} and V^{+}_{I} ordering in the first STEM-ADF image of $FAPbI_3$

After the first STEM-ADF scan at 44 e/Å^2, an ordered vacancy pattern in $Cs_{0.5}FA_{0.5}PbI_3$ is obvious and can be observed directly in the image (Fig. 4A). This is similarly the case for the $FAPbI_3$ in Fig. 1 but far less evident in the $FAPbI_3$ in Fig. 3A. This is due to the lower signal scattered from the organic FA^+ cations in STEM-ADF images, which can be seen from the comparison of Fig. 1A and Fig. 1E. This makes the direct observations of an ordered vacancy patterns in $FAPbI_3$ more challenging, especially when slightly off-axis. In the acquisition of all (S)TEM images, we follow a strict protocol to ensure minimum dose, in particular, all images are taken 'blind', with absolutely NO prior parameter optimization on the QD of interest, including no tilting to zone axis, focusing, adjusting astigmatism etc. This means image acquisition parameters, in particular zone-axis alignment, are often imperfect. Furthermore, the QDs dispersed on the carbon TEM substrate are geometrically unstable and tend to "vibrate" slightly during the acquisition of dose series data. As a result, the ordered vacancy pattern in the STEM-ADF image of $FAPbI_3$ in Fig. 3A (reproduced here in Supplementary Figure 12) is less obvious due to minor mis-orientation from the <001> zone axis. This mis-orientation can be seen from the constant elongation of the "atom shape" into the bottom left direction. However, the intensity line profiles in Supplementary Figure 12(C,D) confirm the existence of the √2 x √2 superstructure of V^{-}_{FA} and V^{+}_{I}.

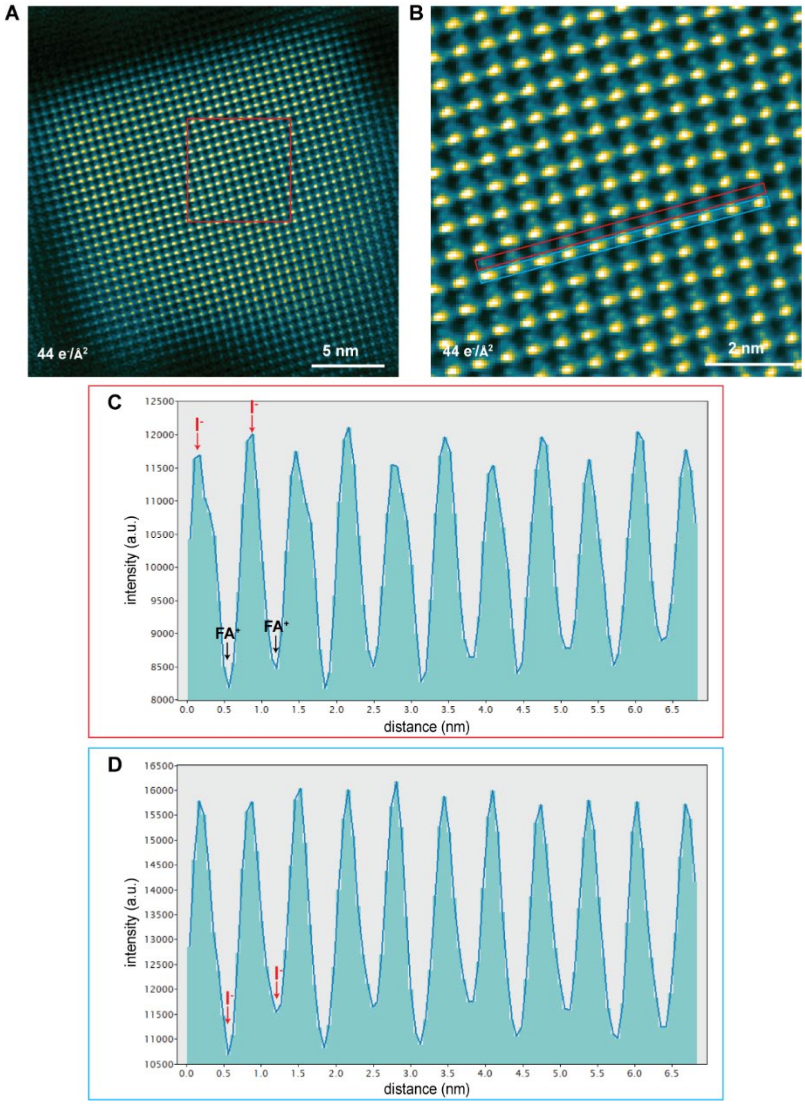

Supplementary Figure 12. Intensity line profiles of the STEM-ADF image of $FAPbI_3$ taken at 44 e/Å^2. (A) first STEM-ADF image (i.e. Fig. 3A). (B) zoom-in image of the region marked in (A). (C) intensity line profile integrated over I^- and FA^+ columns as marked in (B) by red. (C) intensity line profile integrated over Pb^{2+}/I^- and I^- columns as marked in (B) by blue.

Supplementary Note 10

Superstructure fluctuation in the formation of the intermediate phase 2 in $Cs_{0.5}FA_{0.5}PbI_3$

In the STEM-ADF image series of $FAPbI_3$ (Fig. 3), all ${}^{1}/_{2},{}^{3}/_{2},0_c$ family reflections arising from the octahedral tilt phase become evident at 88 e/Å^2. However, a similar STEM-ADF image series of $Cs_{0.5}FA_{0.5}PbI_3$ show fluctuations in the additional reflections (Supplementary Figure 13). The position and intensity of forbidden reflections due to octahedral tilt changes in the first few scans. Only part of the ${}^{1}/_{2},{}^{1}/_{2},0_c$ family of reflections are evident, even until 220 e/Å^2.

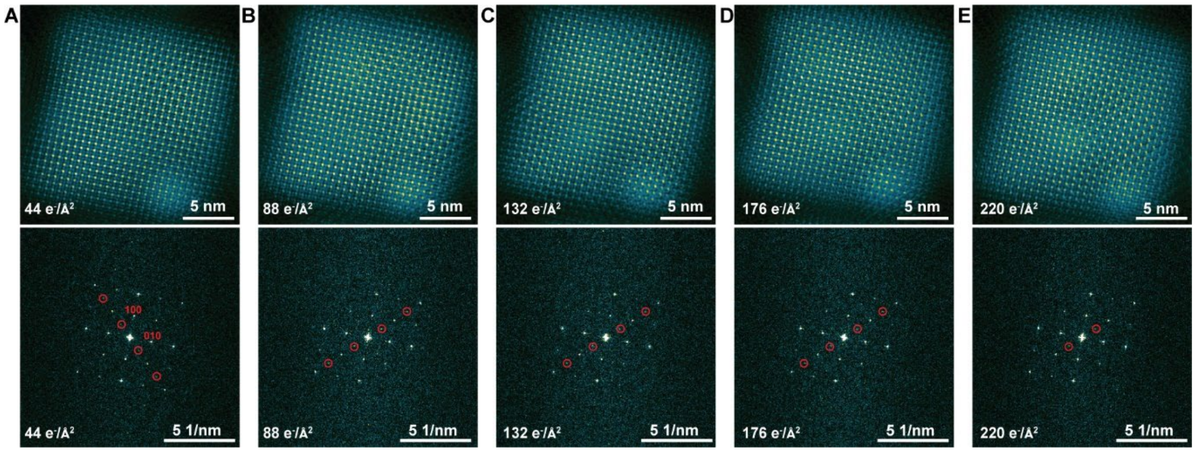

Supplementary Figure 13. Atomic-resolution STEM-ADF images of $Cs_{0.5}FA_{0.5}PbI_3$. (A) 44 e/Å^2. (B) 88 e/Å^2. (C) 132 e/Å^2. (D) 176 e/Å^2. (E) 220 e/Å^2. FTs are given below each image. Additional reflections are marked by red circles.

Supplementary Note 11

STEM-ADF images of $Cs_{0.5}FA_{0.5}PbI_3$ with higher total dose

At a total dose 1280 e/Å^2, strong $^1/_2, ^1/_2, 0_c$ family reflections are evident indicating the formation of an octahedral tilt intermediated phase (Supplementary Figure 14A). However, very weak $^1/_2, ^3/_2, 0_c$ reflections are also observed, as further discussed in supplementary note 11. Furthermore, at a total dose of 2340 e/Å^2, forbidden reflections almost disappear, together with the disappearance of the “flower pattern” in the image (Supplementary Figure 14B). The FT at a total dose of 3400 e/Å^2 shows no forbidden reflections and is consistent with a square perovskite framework, with no octahedral tilt. In addition, the region marked by the red square shows a uniform column image intensity that does not consistent with any phase of the perovskite structure. This will be discussed in detail in supplementary note 12.

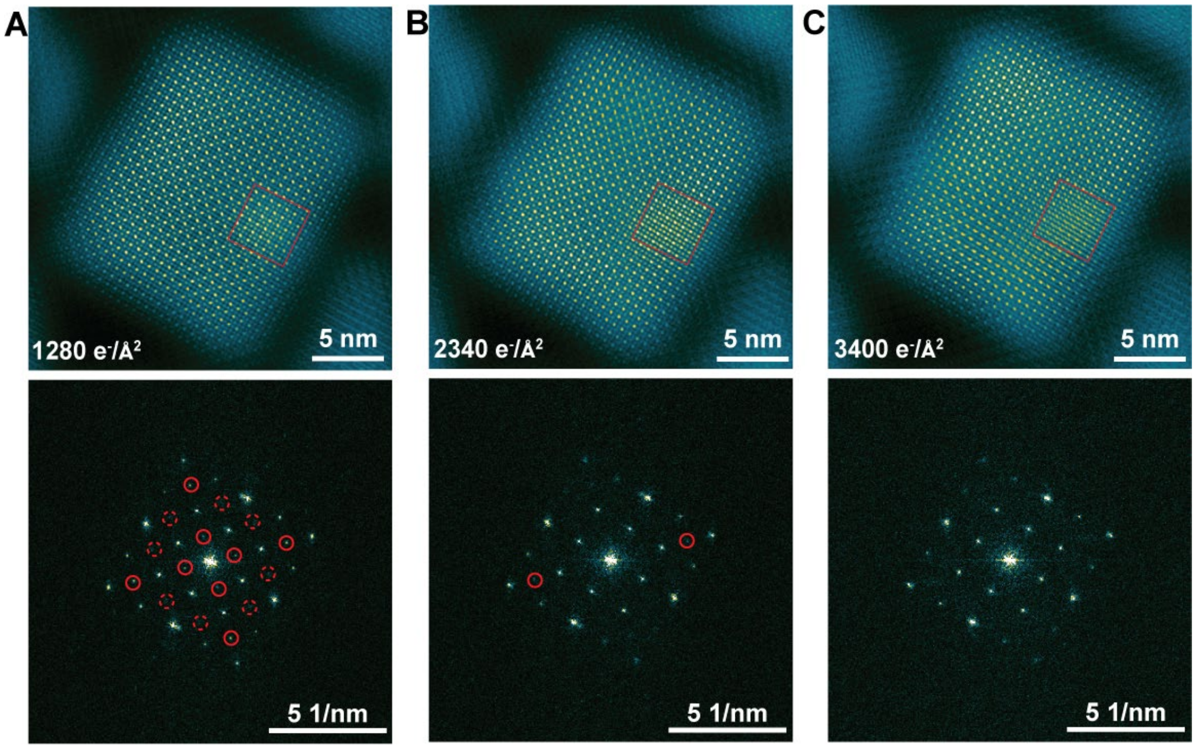

Supplementary Figure 14. STEM-ADF images of a representative $Cs_{0.5}FA_{0.5}PbI_3$ after exposure to a high total dose. (A) 1280 e/Å^2. (B) 2340 e/Å^2. (C) 3400 e/Å^2. FTs are given below each image. Additional reflections are marked by red circles.

Supplementary Note 12

Clusters of PbI_2 and Pb

In the STEM-ADF images taken with the higher levels of total electron dose, clusters with higher intensity can be commonly observed, especially for $Cs_{0.5}FA_{0.5}PbI_3$. We propose two structures to account for these clusters. As shown in Supplementary Figure 15 (same as Fig. 4C), in addition to the reflections that are due to octahedral tilt, we also observe extra reflection at positions that are close to the primary 200_c reflections, but further away from the centre reflection. From an inverse FT (IFT) constructed from those reflections, it is evident they arise from a region with a different lattice structure to that of the perovskite phase (Supplementary Figure 15C). However, for other clusters in this image and also in Supplementary Figure 14, the structure is coherent with the perovskite phase. This suggest these clusters are associated with two different structures.

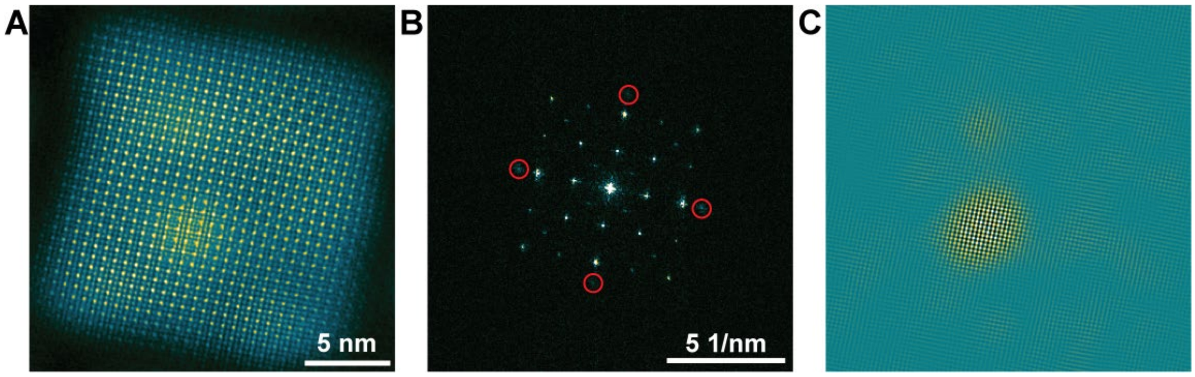

Supplementary Figure 15. STEM-ADF image of $Cs_{0.5}FA_{0.5}PbI_3$ at a total dose of 750 e/Å2 showing higher intensity clusters. (A) STEM-ADF image. (B) FT of (A). (C) IFT of the reflections circled in (B) showing they are associated with a cluster.

The cluster structure that fits coherently with the perovskite lattice is determined to be the PbI_2 phase oriented in the [$\bar{4}$41] direction (Supplementary Figure 16(A, B, D, E, H)). The other cluster structure that is incoherent with the perovskite lattice is found to be pure Pb in the [0001] direction (Supplementary Figure 16(A, C, D, F, G)). It is interesting to note that the coherent PbI_2 occurs more frequently and hence appears to be much more favourable than Pb. In Fig. 4, the incoherent Pb cluster quickly transforms into a coherent PbI_2 cluster, as seen from the lattice image and the disappearance of reflections belonging to Pb (Fig. 4(C, D)).

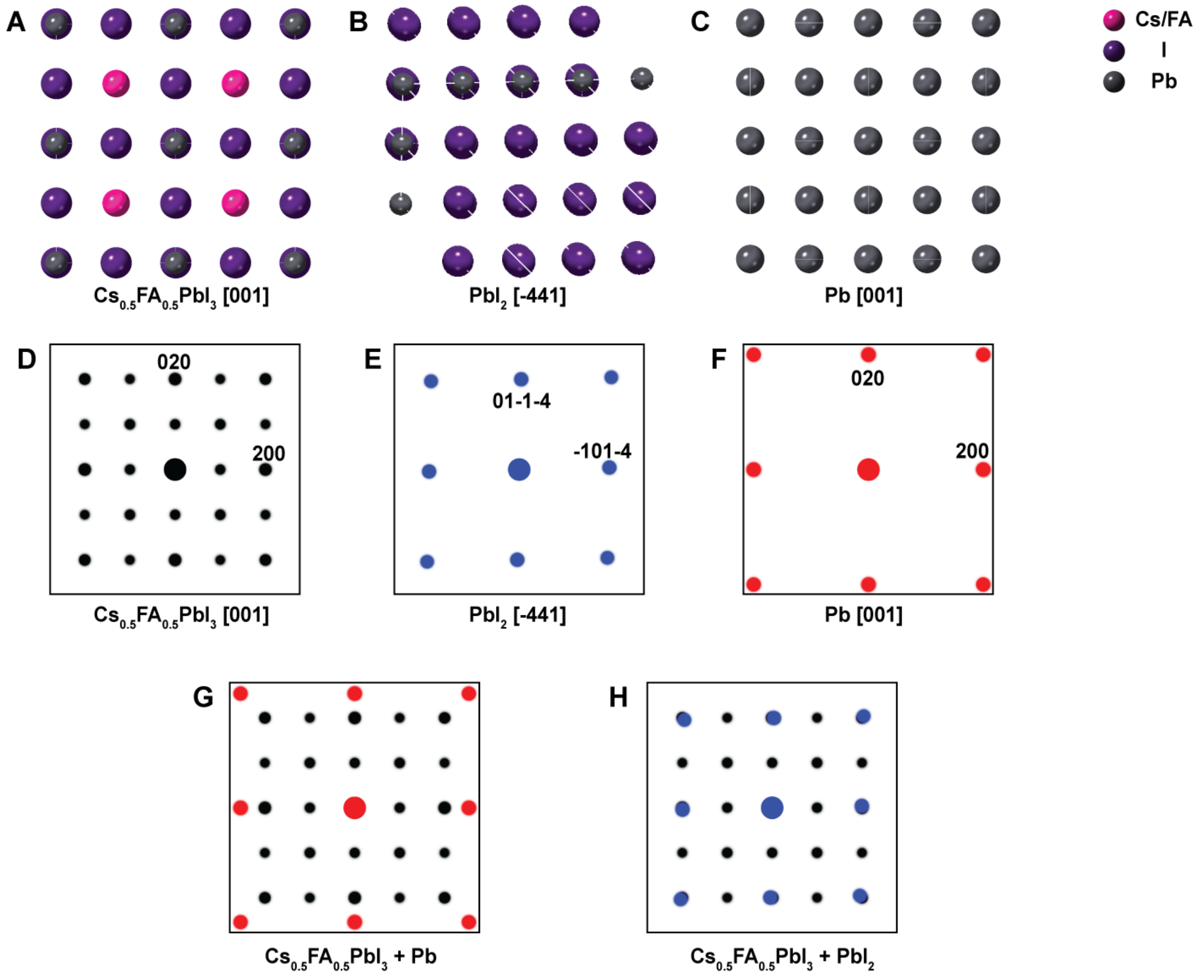

Supplementary Figure 16. Structure of PbI_2 and Pb. (A) Crystal model of cubic perovskite in the [001] projection. (B) Crystal model of PbI_2 in the [$\bar{4}$41] projection. (C) Crystal model of Pb in the [001] projection. (D-F) simulated diffraction patterns of (A-C). (G) overlap of diffraction patterns in (A, F). (H) overlap of diffraction patterns in (A, E).

Once the electron dose exceeds the dose threshold that can be sustained by the perovskite framework, the perovskite structure further decomposes into PbI_2 (Supplementary Figure 17), consistent with previous TEM studies of this system[8] and other perovskite solar cell systems[6,7]. We also notice that the perovskite phase and PbI_2 phase can co-exist in the mixed cation $Cs_{0.5}FA_{0.5}PbI_3$. This could be due to the presence of ad-hoc vacancies in the pre-damaged, pristine structure, so some regions have a 'head-start' towards decomposition over others. Another hypothesis is that there is an inhomogeneous distribution of Cs^+ and FA^+. Given FA^+ vacancies are expected to occur more easily[7], at the electron dose that FA^+ rich regions have decomposed completely into PbI_2, some Cs^+ rich regions still retain the perovskite structure.

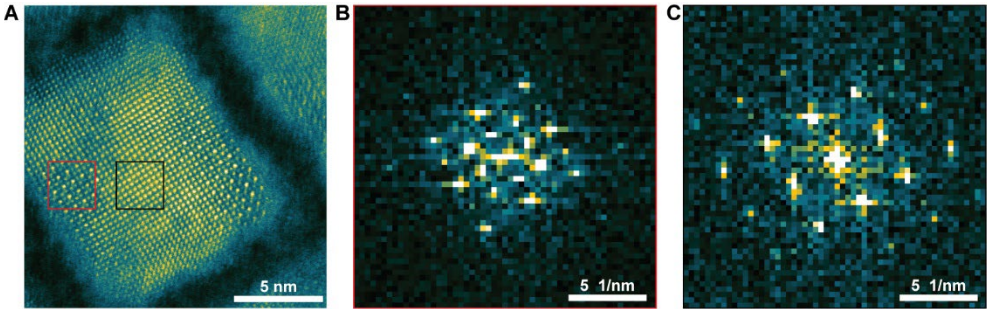

Supplementary Figure 17. Decomposition $Cs_{0.5}FA_{0.5}PbI_3$ into final PbI_2 phase. (A) STEM-ADF image; (B) FT of the region marked in red in (A); (C) FT of the region marked in black in (A).

Supplementary Note 13

Co-existence of two octahedral tilt modes in $Cs_{0.5}FA_{0.5}PbI_3$

We observe two different octahedral modes at the intermediate phase 2. The p4/mbm tetragonal phase is observed primarily in $FAPbI_3$ and the I4/mmm tetragonal phase is only observed in $Cs_{0.5}FA_{0.5}PbI_3$. However, in some $Cs_{0.5}FA_{0.5}PbI_3$, the p4/mbm tetragonal phase is also observed to co-exist with the I4/mmm phase. Interestingly, the FT of an image of a $Cs_{0.5}FA_{0.5}PbI_3$ containing both phases will lead to $1/2, 1/2, 0_c$ and $1/2, 3/2, 0_c$ reflections. These FT reflections are coincident with those from a √2 x √2 superstructure, raising the possibility that the √2 x √2 superstructure could be mistaken for the coexistence of the two octahedral modes and vice versa. However, these two possible scenarios can be distinguished by generating an inverse Fourier transform (IFT) from the $1/2, 1/2, 0_c$ and $1/2, 3/2, 0_c$ reflections separately, as shown in Supplementary Figure 18. This shows that the $1/2, 1/2, 0_c$ and $1/2, 3/2, 0_c$ reflections arise from different (Supplementary Figure 18(B, C)). The corresponding enlarged images from the selected regions confirm that they are two tetragonal phases with different octahedral tilt modes (Supplementary Figure 18(D, E)).

In the TEM studies of OIHPs, the ordered √2 x √2 superstructure of vacancies (intermediate phase 1), in theory, will lead to both $1/2, 1/2, 0_c$ and $1/2, 3/2, 0_c$ forbidden reflections, although they might have very weak intensity. While the subsequent octahedral tilt phase (intermediate phase 2) will result in either $1/2, 1/2, 0_c$ or $1/2, 3/2, 0_c$ forbidden reflections, depending on the octahedral tilt mode. Therefore, extreme care needs to be taken while analysing TEM results, especially when some reflections are missing in SAD patterns or FT patterns.

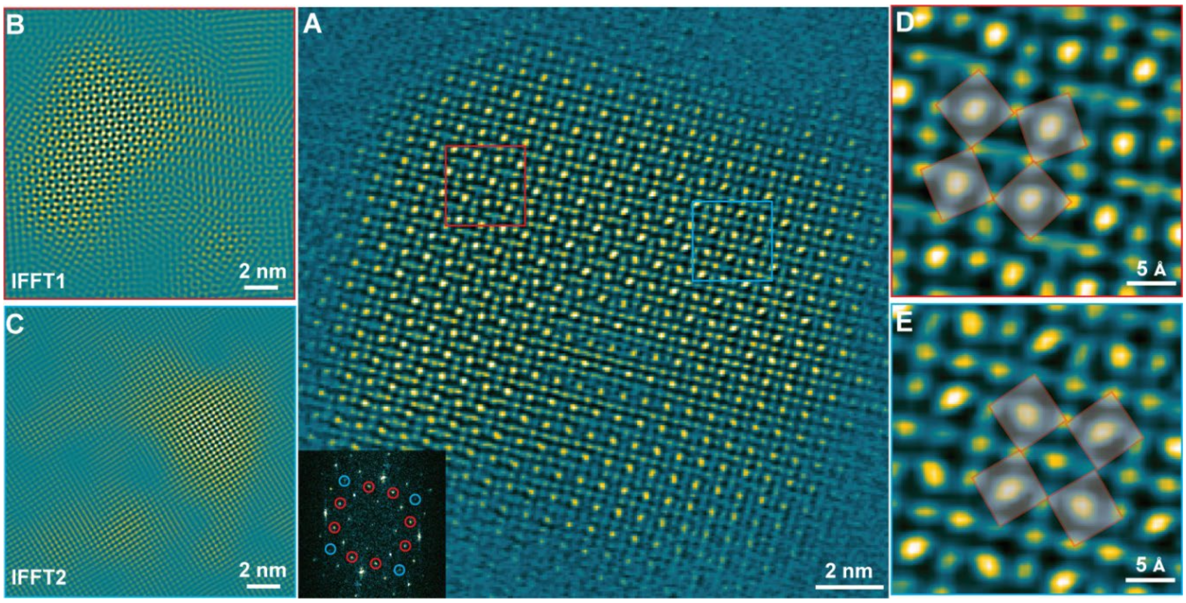

Supplementary Figure 18. STEM-ADF image of $Cs_{0.5}FA_{0.5}PbI_3$ with a total dose of 750 e/Å². (A) Overview STEM-ADF, with FT inserted. (B) IFT from the $1/2, 3/2, 0_c$ reflections circled by red. (B) IFT from the $1/2, 1/2, 0_c$ reflections circled by blue. (D) Enlarged image of region that corresponds to $1/2, 3/2, 0_c$ reflections. (E) Enlarged image of region that corresponds to $1/2, 1/2, 0_c$ reflections.

Supplementary Note 14

Calculations of A-site cation migration energy barrier

The NEB results show that the migration energy barrier of FA in the structure of $FAPbI_3$ is higher than that of Cs in the $CsPbI_3$ structure (Supplementary Figure 19). The figure below shows the migration energy barrier of FA and Cs from 1b crystallographic Wyckoff site to the neighbouring 1b FA-vacancy site and Cs-vacancy site. Compared to FA, Cs has a smaller size, which can be the main reason for the lower migration barrier energy of Cs.

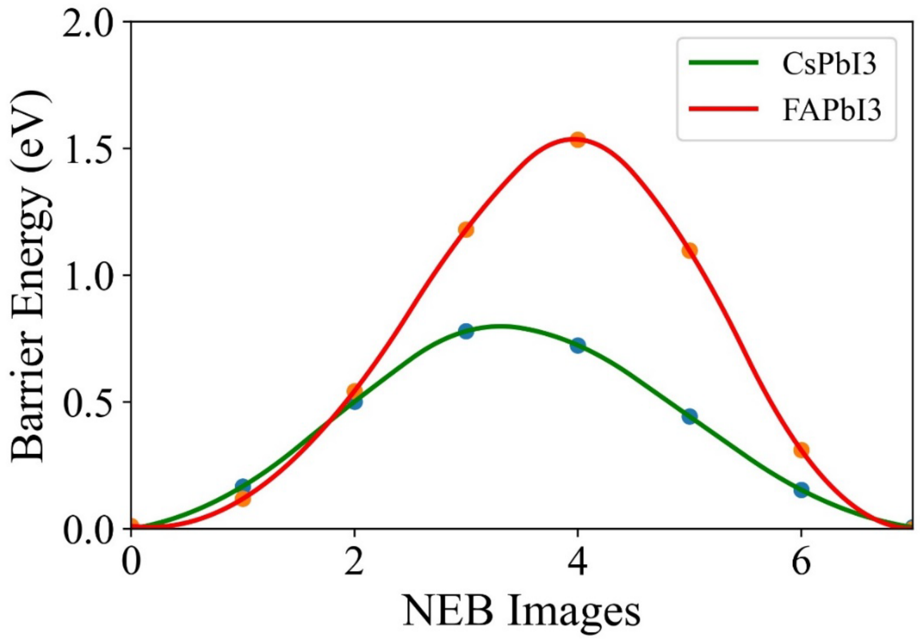

Supplementary Figure 19. NEB results of $FAPbI_3$ and $CsPbI_3$.

Ab-initio molecular dynamics (AIMD) simulations were performed to enhance our understanding of the ionic diffusion mechanisms within the structures of $FAPbI_3$ and $CsPbI_3$. However, conclusive results have not been reached due to the complexity of the system. The aperiodic nature of the structure with initial ad-hoc vacancies, and its subsequent diffusion towards a superlattice, requires an exceptionally large supercell to ensure appropriate periodic boundary conditions. The computational resources required are well-beyond the scope of the present study, however, preliminary, qualitative observations from these simulations can be reported.

For each sample ($FAPbI_3$ and $CsPbI_3$ structures) three trajectories are considered, and each trajectory is simulated for a duration of 20 ps to study the dynamics of Cs atoms and FA molecules. All the calculations were done at 350 K. Based on the results, at the first 10 ps of the trajectories, Cs atoms diffused to the Cs-vacancy site resulting in a change in crystallographic symmetry. However, in none of the $FAPbI_3$ samples did FA exhibit diffusion within the timeframe studied. These results are consistent with the NEB results and show that, *for a given vacancy concentration*, Cs diffusion in $CsPbI_3$ is easier than FA diffusion in $FAPbI_3$. In practice, the vacancy concentration also needs to be taken into consideration. The vacancy concentration is known to affect the ionic diffusion of elements. Typically, a higher concentration of vacancies tends to lower the energy barrier for ionic diffusion. This is also observed here in the AIMD simulations. In reality, $FAPbI_3$ is expected to have significantly more vacancies compared with $CsPbI_3$. This is because FA readily decomposes into smaller molecules (such as NH_3 and CH_2N) and evaporates, facilitating vacancy formation, particularly in the presence of stimuli such as light, heat and electron beams[9]. This is consistent with our experimental observations which suggest that more FA vacancies form in $FAPbI_3$, compared with A-site vacancies in $FA_{0.5}Cs_{0.5}PbI_3$, for a given electron dose (see main manuscript). This higher vacancy concentration for FA is likely to lead to a reduction in the energy barrier for FA migration.

Appendix

Raw images and filtering for each image

Raw/Filtered Low-dose STEM-ADF images in Fig. 1, Fig. 2 Fig. 3 and Fig. 4 from the main text are summarized in the appendix. A Bragg-Butterworth filter[10,11] has been applied to these images as detailed in Supplementary Figure 4B.

Figure 1

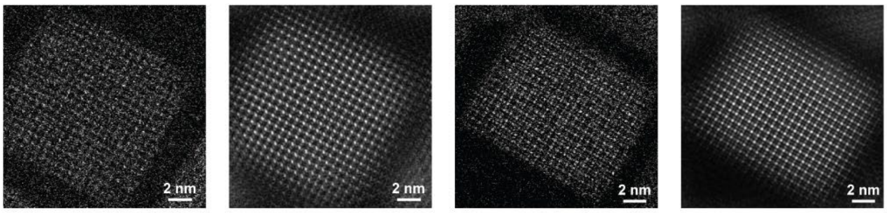

Figure 2

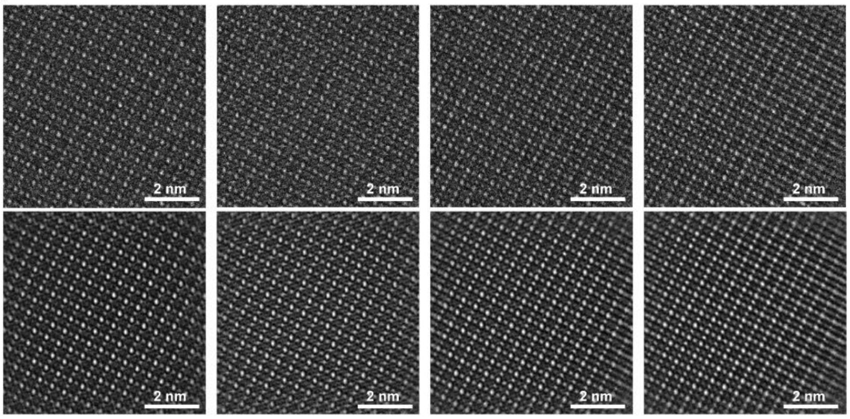

Figure 3

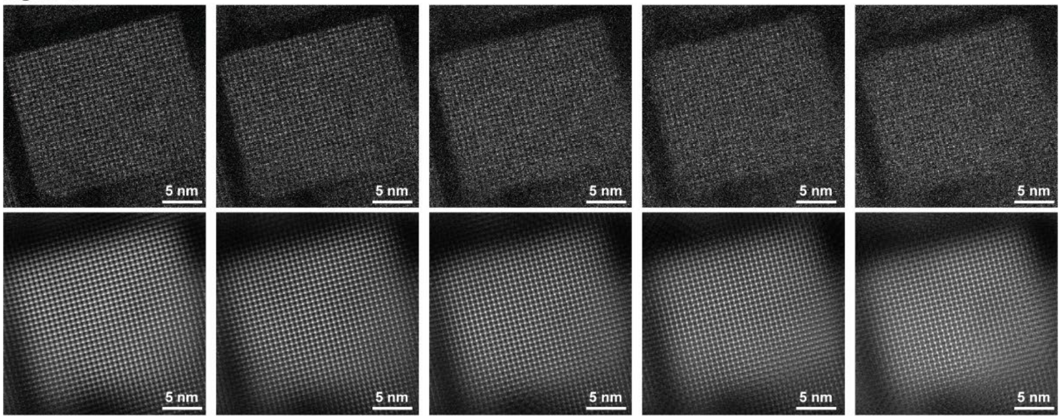

Figure 4

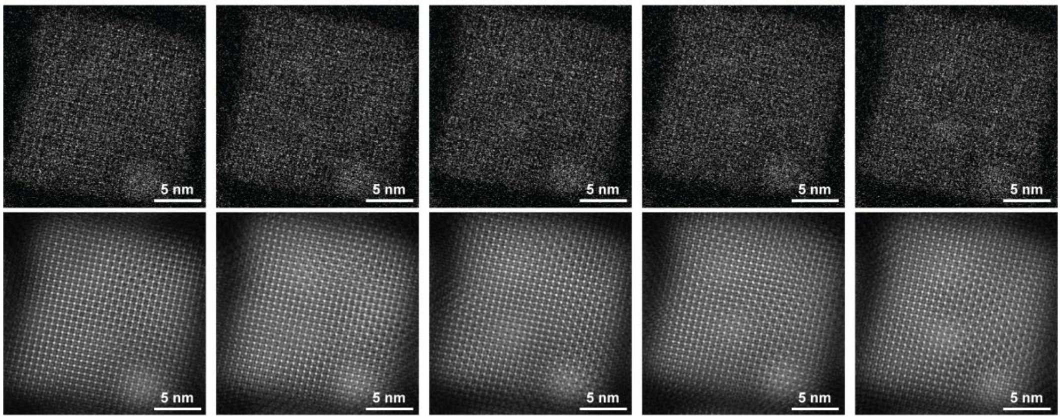

Supplementary References

(1) Krause, F. F.; Schowalter, M.; Oppermann, O.; Marquardt, D.; Müller-Caspary, K.; Ritz, R.; Simson, M.; Ryll, H.; Huth, M.; Soltau, H.; Rosenauer, A. Precise Measurement of the Electron Beam Current in a TEM. *Ultramicroscopy* **2021**, *223*, 113221.

(2) Tate, M. W.; Purohit, P.; Chamberlain, D.; Nguyen, K. X.; Hovden, R.; Chang, C. S.; Deb, P.; Turgut, E.; Heron, J. T.; Schlom, D. G.; Ralph, D. C.; Fuchs, G. D.; Shanks, K. S.; Philipp, H. T.; Muller, D. A.; Gruner, S. M. High Dynamic Range Pixel Array Detector for Scanning Transmission Electron Microscopy. *Microsc. Microanal.* **2016**, *22* (1), 237–249.

(3) Nguyen, D. T.; Findlay, S. D.; Etheridge, J. The Spatial Coherence Function in Scanning Transmission Electron Microscopy and Spectroscopy. *Ultramicroscopy* **2014**, *146*, 6–16.

(4) Nguyen, D. T.; Findlay, S. D.; Etheridge, J. A Menu of Electron Probes for Optimising Information from Scanning Transmission Electron Microscopy. *Ultramicroscopy* **2018**, *184*, 143–155.

(5) Wu, X.; Ke, X.; Sui, M.; Ke, X. Recent Progress on Advanced Transmission Electron Microscopy Characterization for Halide Perovskite Semiconductors. *J. Semicond.* **2022**, *43* (4), 041106.

(6) Rothmann, M. U.; Kim, J. S.; Borchert, J.; Lohmann, K. B.; Leary, C. M. O.; Sheader, A. A.; Clark, L.; Snaith, H. J.; Johnston, M. B.; Nellist, P. D.; Herz, L. M. Atomic-Scale Microstructure of Metal Halide Perovskite. *Science* **2020**, *370*.

(7) Chen, S.; Wu, C.; Han, B.; Liu, Z.; Mi, Z.; Hao, W.; Zhao, J.; Wang, X.; Zhang, Q.; Liu, K.; Qi, J.; Cao, J.; Feng, J.; Yu, D.; Li, J.; Gao, P. Atomic-Scale Imaging of CH3NH3PbI3 Structure and Its Decomposition Pathway. *Nat. Commun.* **2021**, *12* (1), 1–7.

(8) Hao, M.; Bai, Y.; Zeiske, S.; Ren, L.; Liu, J.; Yuan, Y.; Zarrabi, N.; Cheng, N.; Ghasemi, M.; Chen, P.; Lyu, M.; He, D.; Yun, J. H.; Du, Y.; Wang, Y.; Ding, S.; Armin, A.; Meredith, P.; Liu, G.; Cheng, H. M.; Wang, L. Ligand-Assisted Cation-Exchange Engineering for High-Efficiency Colloidal Cs1−xFAxPbI3 Quantum Dot Solar Cells with Reduced Phase Segregation. *Nat. Energy* **2020**, *5* (1), 79–88.

(9) Protesescu, L.; Yakunin, S.; Bodnarchuk, M. I.; Bertolotti, F.; Masciocchi, N.; Guagliardi, A.; Kovalenko, M. V. Monodisperse Formamidinium Lead Bromide Nanocrystals with Bright and Stable Green Photoluminescence. *J. Am. Chem. Soc.* **2016**, *138* (43), 14202–14205.

(10) Dogra, A.; Bhalla, P. Image Sharpening by Gaussian and Butterworth High Pass Filter. *Biomed. Pharmacol. J.* **2014**, *7* (2), 707–713.

(11) Kirkland, E. J.; Siegel, B. M.; Uyeda, N.; Fujiyoshi, Y. Improved High Resolution Image Processing of Bright Field Electron Micrographs. II. Experiment. *Ultramicroscopy* **1985**, *17* (2), 87–103.
